# SIGNALING EFFICACY DRIVES THE EVOLUTION OF LARGER SEXUAL ORNAMENTS BY SEXUAL SELECTION

**DOI:** 10.1111/evo.12255

**Published:** 2013-09-30

**Authors:** Samuel J Tazzyman, Yoh Iwasa, Andrew Pomiankowski

**Affiliations:** 1 Theoretical Biology, Institute of Integrative BiologyETH Zürich, Universitätstrasse 16, CH-8092, Zürich, Switzerland; 2 CoMPLEX, University College LondonGower Street, London, WC1E 6BT, United Kingdom; 3 The Galton Laboratory Department of Genetics Environment, and Evolution, University College LondonGower Street, London, WC1E 6BT, United Kingdom; 5 Department of Biology Faculty of Sciences, Kyushu UniversityFukuoka 812-8581, Japan

**Keywords:** Fisher's runaway, mate choice, mate preference, sexual dimorphism, sexual ornament, sexual selection

## Abstract

Why are there so few small secondary sexual characters? Theoretical models predict that sexual selection should lead to reduction as often as exaggeration, and yet we mainly associate secondary sexual ornaments with exaggerated features such as the peacock's tail. We review the literature on mate choice experiments for evidence of reduced sexual traits. This shows that reduced ornamentation is effectively impossible in certain types of ornamental traits (behavioral, pheromonal, or color-based traits, and morphological ornaments for which the natural selection optimum is no trait), but that there are many examples of morphological traits that would permit reduction. Yet small sexual traits are very rarely seen. We analyze a simple mathematical model of Fisher's runaway process (the null model for sexual selection). Our analysis shows that the imbalance cannot be wholly explained by larger ornaments being less costly than smaller ornaments, nor by preferences for larger ornaments being less costly than preferences for smaller ornaments. Instead, we suggest that asymmetry in signaling efficacy limits runaway to trait exaggeration.

How the flamboyant ornamental traits used to attract mates in various species evolve is a question that has been debated since Darwin's time (Cronin 1991). There are many theoretical models suggesting how preferences and ornaments coevolve (reviewed by Mead and Arnold 2004; Kuijper et al. 2012). However, there is an overlooked difficulty with these models. They either lack an explicit directionality for the exaggerated trait, or predict that secondary sexual ornaments should be just as likely to evolve to be smaller (hereafter referred to as “reduced” traits) as to be larger (hereafter referred to as “exaggerated” traits) than the natural selection optimum.

In his original setting out of the runaway process, Fisher referred to sexual preferences being for a “plumage character” (Fisher 1930). His verbal framework was based upon an initial majority of females with a particular preference. This arbitrary initial preference eventually causes a runaway, but there is no reason why it should be toward exaggerated rather than reduced traits (Dawkins 1986, p. 215). This symmetry between exaggeration and reduction is seen in the classic mathematical models of the Fisher's process (Lande 1981). Under this framework, upon which many other models are based (see Mead and Arnold 2004; Kuijper et al. 2012), there is a line of equilibrium. If this line is stable, then any ornamental trait value corresponds to an equilibrium point (and thus reduced and exaggerated traits are equally likely). If the line is unstable, then runaway (in the sense of perpetual evolution away from the line of equilibrium) is equally likely in either direction. Alternatively, some models only allow trait evolution in one direction, so that there is either “trait” (possibly of various sizes) or “no trait” (Kirkpatrick 1996; Kuijper et al. 2012). This single direction of evolution could represent reduction just as well as exaggeration, however, so it does not answer our problem.

This implicit balance between exaggeration and reduction is present in a number of variants of the Fisherian model of sexual selection (Mead and Arnold 2004; Kokko et al. 2006; Kuijper et al. 2012), including major gene models (Kirkpatrick 1982), quantitative genetic models (Lande 1981; Iwasa and Pomiankowski 1995; Tazzyman and Iwasa 2010), models with spatial structure (Kirkpatrick 1982), and those for multiple traits (Pomiankowski and Iwasa 1993). Fisher's runaway also can be accompanied by the handicap principle (Zahavi 1975). Under this framework, ornaments must be costly to their bearers, and this cost must be higher for lower quality bearers, so a large ornament can be reliably adjudged to be underpinned by “good genes” or other forms of quality (Grafen 1990; Iwasa et al. 1991; Iwasa and Pomiankowski 1999). However it is not only exaggerated phenotypic traits that will be costly. Traits smaller than the natural selection optimum will also be likely to handicap their bearer. So *a priori*, the additional force of the handicap principle seems just as likely to evolve reduced as exaggerated traits (e.g., Pomiankowski 1987; Iwasa et al. 1991; Iwasa and Pomiankowski 1994; Kirkpatrick 1996; Pomiankowski and Iwasa 2001).

So does the theoretically predicted equality between exaggeration and reduction actually exist in the natural world? A previous literature review of preference in acoustic cues found that “if females prefer traits that deviate from the population mean, they usually prefer traits of greater quantity” (Ryan and Keddy-Hector 1992), suggesting that the evolution of exaggerated ornamentation is favored over the evolution of reduced ornamentation. If this is true, then there must be something missing from the theory. Several possibilities suggest themselves from the structure of sexual selection models. One conceivable explanation is that female choice for exaggerated ornaments is less costly. It is well known that the introduction of costs to female preferences affects the equilibrium line in classic quantitative genetics models of Fisher's runaway, in many cases reducing it to a point or a group of points (Mead and Arnold 2004; Kuijper et al. 2012). If it is less costly for females to prefer males with exaggerated rather than reduced ornaments, this may promote the evolution of exaggeration. A second possibility is that exaggerated ornaments themselves are less costly than reduced ornaments. Although the cost of an ornament must increase as its size deviates from the natural selection optimum, the rate at which this cost increases need not be the same for exaggerated as for reduced traits. A third potential explanation is that the signaling efficacy of an ornament increases with its size. This will be the case if it is easier for females to perceive exaggerated traits than reduced traits, particularly from a distance (or if they can perceive differences in traits more easily when traits are exaggerated). It may also be true that larger traits are more reliable as signals. All of these possibilities seem biologically reasonable. But they need to be examined theoretically to test their plausibility as explanations for the lack of equality between exaggeration and reduction.

An alternative fourth possibility is that the types of traits preferred by females do not admit the possibility of exaggeration. For these traits, the natural selection optimum is no trait at all, and the trait therefore functions purely as a sexual ornament. This case has been considered previously in models in which only one direction of exaggeration is possible (e.g., Kirkpatrick 1982), and additionally in a polygenic model (Supplementary Material to Kokko et al. 2006).

Our study takes two parts. First, we carry out a survey of articles about mate preference to investigate whether there are examples of reduced ornaments and if so, whether they are associated with particular types of ornamental traits. Second, we use a simple mathematical model based on a classic modeling framework (Pomiankowski and Iwasa et al. 1991) to investigate if any of the three possible explanations above can explain in principle why the evolution of exaggerated traits might be more likely than the evolution of reduced traits.

## Are There Any Reduced Traits?

### LITERATURE REVIEW

We searched the literature on secondary sexual ornamentation using the Web of Knowledge, with the search terms “sexual selection” and “sexual ornament,” between 1993 and 2011 (since the review in Ryan and Keddy-Hector 1992). We only included articles with experimental or field evidence of mate preference for a particular phenotypic trait. We excluded articles in which the object of female mate preference was not a distinct signaling trait, such as those in which females favored aerobic capacity, condition, immunocompetence, age, or symmetry. We measured the relative frequency of reduced versus exaggerated ornamentation across this sample. Our search criteria gave us 148 articles, spanning a wide variety of taxa (97 species), with diverse attractive traits (Supplementary Table S1). The ornamental traits are discussed under four headings: color, behavior, pheromones, and morphology. Note that our objective was to make a reasonable sample of the field, not to fully survey all work in this area.

#### (a) Color

There was a high diversity of colors identified as sexually attractive across the full range of taxa (Supplementary Table S1). Color traits included patches on particular body parts (e.g., Red Junglefowl *Gallus gallus* [Johnson et al. 1993; Zuk et al. 1995a], stickleback *Gasterosteus aculeatus* [Bakker 1993; Kraak et al. 1999]) and general body colors (e.g., Canary *Serinus canaria* [Heindl and Winkler 2003], Cabbage Butterfly *Pieris rapae* [Morehouse and Rutowski 2010]), including cases in which the amount of ultraviolet reflectance was important (e.g., budgerigars *Melopsittacus undulatus* [Zampiga et al. 2004; Griggio et al. 2010], King Penguins *Aptenodytes patagonicus* [Nolan et al. 2010]).

The topology of the phenotypic space for color is not obviously translatable to a single dimension used for ornament evolution in theoretical models. How should exaggeration and reduction be defined in this space? One possibility would be to consider the size of the color patch as the ornament, with larger patches being exaggerated and smaller ones being reduced. This requires that the natural selection optimum is to have a certain size of patch, typically what is seen in females (assuming that females represent the natural selection optimum). If females lack the trait entirely, it is harder to consider that preference for reduced patch size could evolve. Another possibility is to define a particular color, for example, red, as being exaggeration, and then define its “opposite,” green, as reduction, with cryptic coloration being the natural selection optimum (Iwasa and Pomiankowski 1994). Related to this might be a dimension of brighter/darker coloration, as there were examples of both preferences for brighter plumage (e.g., kestrel *Falco tinnunulus* [Palokangas et al. 1994]) and for darker plumage (e.g., Pied Flycatcher *Ficedula hypoleuca* [Canal, Potti, and Davila 2011; Galvan and Moreno 2009]). This preserves the possibility that there are reduced as well as exaggerated states. However, in general it seems somewhat contrived to construct a simple one-dimensional scale of exaggeration/reduction “opposites.”

The plethora of different preferences seen suggest that coloration lies on a higher dimensional equivalent of the exaggerated/reduced dichotomy discussed above, and sexually selected runaway can occur in a number of directions. Evidence for this possibility is indicated by diverse preferences for color markings in different populations of the same species (e.g., minnow *Phoxinus phoxinus* [Kekalainen et al. 2010], guppy *Poecilia reticulata* [McKinnon 1995; Gong and Gibson 1996; Brooks and Couldridge 1999]). Care must be taken in drawing conclusions however, as there is evidence that females simply prefer color patterns that are rarely seen (Olendorf et al. 2006; Hampton et al. 2009; Johnson et al. 2010).

Some examples suggest that different populations have opposite preferences for exaggerated and reduced traits. In House Sparrows *Passer domesticus*, some populations have females preferring males with smaller black throat “badges” in a species in which generally preference is for larger badge size (Simon et al. 1999). Likewise in the eastern mosquitofish *Gambusia holbrooki*, some populations prefer males with more melanic spots whereas others prefer males with fewer melanic spots (Bisazza and Pilastro 2000). These examples show that it is possible for preference for reduced ornaments to exist on a local scale. They leave open the question why reduced preferences have not driven the loss of the male ornamental trait. It has been suggested that preferences are plastic and vary with local circumstances, reflecting differences in the reproductive value of male with reduced or exaggerated traits (Simon et al. 1999). In conclusion, it may be possible to define a direction in color space as being exaggerated and another direction as being reduced for a given species, but as a general rule this seems unlikely beyond a few specific cases.

#### (b) Behavior

Specific displays involved in courtship are often important in mate preference. There was evidence for the attractiveness of increased rates or intensity of courtship behavior, calls, or songs in several bird species (Red junglefowl *Gallus gallus* [Zuk et al. 1995b; Chappell et al. 1997; Wilson et al. 2008], Barn Swallow *Hirundo rustica* [Moller et al. 1998], Spotted Bowerbird *Chlamydera maculata* [Borgia and Presgraves 1998], Pheasant *Phasianus colchicus* [Mateos and Carranza 1999], Gambel's Quail *Callipepla gambelii* [Hagelin and Ligon 2001], Peafowl *Pavo cristatus* [Loyau et al. 2005], Hooded Warbler *Wilsonia citrina* [Chiver et al. 2008]), and in several nonbird species (fruit fly *Drosophila grimshawii* [Droney 1996; Droney and Hock 1998], wolf spiders *Hygrolycosa rubrofasciata* [Kotiaho et al. 1996] and *Schizocosa stridulans* [Hebets et al. 2011], poison frogs *Dendrobates leucomelas* and *Epipedobates tricolor* [Forsman and Hagman 2006], guppy *Poecilia reticulata* [Nicoletto 1993; Kodric-Brown and Nicoletto 2001]). Again it is difficult to see how reduced ornaments are possible in this situation. In most cases, the natural selection optimum presumably is the absence of any courtship behavior at all, which leaves no or little possibility for reduction. In behavioral traits we conclude that reduction is very unlikely or impossible.

#### (c) Pheromone

There were three examples in our sample in which preference was for odors (fruit fly *Drosophila grimshawi* [Droney and Hock 1998], minnow *Phoxinus phoxinus* [Kekalainen et al. 2011], Iberian rock lizard *Iberolacerta cyreni* [Martin and Lopez 2008]). With pheromones, it is again difficult to see how reduced traits are possible. The chemical space of pheromones permits runaway in several directions, similar to the possibilities for colors discussed above. It may be that in some cases the pheromone is a natural by-product of some essential process, and thus reduction and exaggeration of its quantity could be defined. But in general, the concept of a reduced pheromone trait is not one that is easily tractable.

#### (d) Morphology

The final group we discuss contains morphological traits subject to sexual selection, in which the size of the trait is not optimal from the perspective of survival. There were many examples of species in which there is preference for exaggerated traits (see Supplementary Table S1 for details). Of the 40 examples of species in which morphological traits were subject to mating preference, 34 were unambiguously for exaggerated traits. The remaining six provide evidence for the possibility of preference for reduced traits. In stalk-eyed flies (*Teleopsis dalmanni*), artificial selection for males with shorter eyespan was associated with the evolution of female preference for shorter eyespan (Wilkinson and Reillo 1994), in contrast to the usual preference for longer eyespan (Wilkinson and Reillo 1994; Hingle et al. 2001). In *Xiphophorus* swordtail species, the presence of a longer tail fin (the “sword”) is usually attractive to females (Rosenthal and Evans 1998; Rosenthal et al. 2001; Johnson and Basolo 2003). But in some cases, there was evidence that smaller swords were preferred. In *X. helleri*, this was the case after females had been exposed to a predator (Johnson and Basolo 2003), or had only ever been exposed to short-sworded males (Walling et al. 2008). This context-dependent preference shows the possibility for preferences for reduced traits. In *X. birchmanni*, males have lost their ancestral swords, and females find swordless males more attractive (Wong and Rosenthal 2006), suggesting that female preference for reduced traits has contributed to the evolutionary loss of the male ornament (although we found no examples of males with smaller tail fins than females, i.e., “antiswords,” for want of a better term). Another study found evidence that female preference for dorsal fin length is disruptive, with females preferring both shorter and longer dorsal fins compared to those that are average size (Robinson et al. 2011). In birds, for both the golden-headed cisticola *Cisticola exilis* and the fairywren *Malurus melanocephalus*, males with smaller tails seemed to be preferred by females. However in both cases, there were possible mitigating factors that mean this may not be as simple as female preference for reduced tail size. In the case of the cisticola, females may in fact be choosing males for their aerodynamic ability, which is improved by a shortened tail (Balmford et al. 2000) whereas in the fairywren, shortened tails seemed to be a signal that affected male–male competition rather than female choice (Karubian et al. 2009).

Of the examples of preference for exaggerated traits, there were some cases in which no reduction of trait size was possible. In the wolf spider, *Schizocosa crassipes*, males (but not females) sported ornamental “leg tufts,” for which female preference was shown (Hebets and Uetz 2000) and in the Mexican molly, *Poecilia sphenops*, females preferred males who had a moustache-like growth on their upper lip, with no such trait noted as existing on females (Schlupp et al. 2010). For birds, there were three examples of species in which preferred males bore larger ornamental traits of a type apparently absent in females: snoods and skullcaps in wild turkeys, *Meleagris gallopavo* (Buchholz 1995); ear tufts in ring-necked pheasants, *Phasianus colchichus* (Mateos and Carranza 1995); and combs in Red Junglefowl, *Gallus gallus* (Johnson et al. 1993; Zuk et al. 1995a; Johnsen and Zuk 1996; Ligon et al. 1998; Cornwallis and Birkhead 2007). However in the vast majority of cases, preference was for exaggerated forms of morphological traits also possessed by the female, such as fins, wings, and tails, among many other examples (Delope and Moller 1993; Jones and Hunter 1993; Moller 1993a, b1993b; Petrie and Williams 1993; Macias et al. 1994; Oakes and Barnard 1994; Wilkinson and Reillo 1994; Simmons 1995; Weatherhead and Boag 1995; Goddard and Mathis 1997; Karino 1997; Saino et al. 1997; Yezerinac and Weatherhead 1997; Marchetti 1998; Oliveira and Custodio 1998; Rosenthal and Evans 1998; Tomkins and Simmons 1998; Wilkinson et al. 1998; Jones and Hunter 1999; Kraak et al. 1999; Hagelin and Ligon 2001; Pryke et al. 2001; Regosin and Pruett-Jones 2001; Rosenthal et al. 2001; Velando et al. 2001; Calkins and Burley 2003; Daunt et al. 2003; Hagelin 2003; Johnson and Basolo 2003; Okuda et al. 2003; Veit and Jones 2003; Candolin 2004, 2005; McGlothlin et al. 2005; Pryke and Andersson 2005; Cotton et al. 2006; Moreno-Rueda 2006; Murphy 2007; Malmgren and Enghag 2008; Pizzolon et al. 2008; Sirkia and Laaksonen 2009; Watson and Simmons 2010; Canal et al. 2011; Karino et al. 2011; Robinson et al. 2011; South and Arnqvist 2011 see Supplementary Table S1 for details). In these cases, reduction would be possible.

#### (e) Summary

We conclude that preference for reduced ornamentation can exist, but examples are very scarce. In part, this is because many sexual ornaments simply do not exist on a simple reduced/exaggerated scale, but rather are bounded by a natural selective optimum of no trait. This relates to most color, behavior, and pheromone ornaments, and a few morphological traits. However, we found a large number of cases in which reduced ornamentation would be possible, because the sexually selected trait is a morphological trait that could be either reduced or exaggerated in size from the natural selective optimum. Overwhelmingly in such cases exaggeration evolves rather than reduction.

The few potential cases of reduced ornamentation that we found in the literature were not clear-cut. In particular, preferences for reduced ornaments were often context dependent, probably being associated with avoidance of dangerous behavior by the female. Alternatively, the reduced male ornament was not directly preferred, but rather led to a better display that itself was the object of female preference. We conclude that the predicted equality between exaggeration and reduction in ornamentation is not observed, a finding that echoes the previous review by Ryan and Keddy-Hector (Ryan and Keddy-Hector 1992).

It may be that there are good examples in the natural world of reduced ornamental traits that have simply not been explored yet (or have been missed by our survey). Certainly it seems likely that by their very nature, reduced traits would be less apparent, and so less likely to become items of interest and study. There also might be a greater difficulty in species in which there are multiple ornaments (of which there are many, Supplementary Table S1). The presence of an exaggerated trait in such species might make it difficult to notice the reduced trait alongside it. But even if there are examples of reduced ornamental traits yet to be discovered or reported, it seems unlikely that they will be uncovered in such numbers as to satisfy the theoretically predicted equality between exaggeration and reduction. We, therefore, conclude that there must be important differences between these two types of traits that existing models of sexual selection do not capture. It is to these differences we turn to consider how they might make the evolution of reduced secondary sexual ornamentation much less likely than the evolution of exaggerated secondary sexual ornamentation.

## Model

Because Fisher's runaway occurs alongside all hypotheses of the evolution of sexual selection, it is the ideal starting point for a theoretical analysis of exaggeration and reduction in sexual selection. It has been described previously as a null model for sexual selection (Kirkpatrick and Ryan 1991; Kuijper et al. 2012). We extend a classic model of Fisherian runaway (Pomiankowski et al. 1991; Pomiankowski and Iwasa 1993) to explore whether asymmetry in the cost of preference, the cost of the male ornament, or signaling efficiency could explain why reduced traits are less likely to evolve (the three possibilities outlined in the Introduction).

We model the evolution of male ornament size (*t*) and female preference (*p*) using a two-trait sexual selection model (adapted from [Pomiankowski et al. 1991]). Both traits are assumed to have a polygenic, additive genetic basis, and we assume weak selection. The change in mean phenotype per generation is modeled as


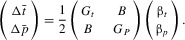
(1)

The terms 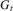 and 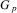 are the additive genetic variances for male ornament size and female preference, respectively, and are assumed to be constant. The term *B* is the genetic covariance between the two traits. The 1/2 coefficient is due to the sex-limited expression of the traits. The terms 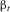 and 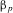 are the selection gradients on male ornament size and female preference, respectively.

We define the following fitness functions for males and females, respectively:

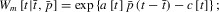
(2a)

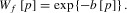
(2b)

Equation (2a) gives the expected fitness of a male with ornament size *t* in a population with mean ornament size 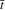 and mean female preference 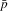. Within the curly braces on the right-hand side, there are two terms. The first term describes the effect of sexual selection on the male. This is a product of the average female preference 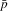, the difference between the male's ornament and the average ornament 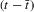, and the signaling efficacy of the male's ornament, *a*[*t*]. If mean female preference is positive, males with ornaments larger than the mean will benefit via sexual selection, and conversely, if mean female preference is negative, males with ornaments smaller than the mean will benefit. The size of this benefit is controlled by the efficacy of signal, *a*[*t*], which is a function of ornament size *t*. Signaling efficacy cannot be zero or negative, so *a*[*t*] > 0 for all *t*. The second term in curly braces on the right-hand side of equation (2a) describes the natural selection cost of bearing the ornament. It is again a function of *t*, *c*[*t*]. It must be nonnegative, and we assume that it reaches a minimum at the natural selection optimum, which we assign to *t* = 0, so that 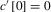. The cost of an ornament increases as it deviates from *t* = 0, as does the rate of increase of this cost (i.e., *c*[*t*] is convex).

Equation (2b) gives the expected fitness of a female with preference *p*. In the curly braces on the right-hand side is the function *b*[*p*]. This is the cost to a female of having a preference *p*. It must be nonnegative *b*[*p*] ≥ 0 for all *p*, and we assume that it has a minimum at *p* = 0, corresponding to no preference when mating, so that 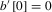. Values of *p* < 0 indicate a preference for males whose ornaments are smaller than the population mean, and values of *p* > 0 indicate a preference for males whose ornaments are larger than the population mean. The further from *p* = 0 in either direction, the stronger the preference, and the higher the cost (we assume *b*[*p*] is convex). Because female choice is assumed to be less costly than bearing an ornament, we assume that *b*[*p*] << *c*[*t*].

The selection gradients are then determined from the fitness equations above as follows:

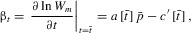
(3a)

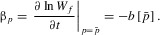
(3b)

The only point for which 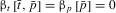 is 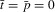. Using standard quantitative genetics techniques (Barton and Turelli 1991; Pomiankowski and Iwasa 1993), we can establish that the genetic covariance *B* will be approximately equal to


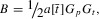
(4)

or 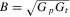, whichever is the smaller (Appendix 1). We can then substitute this, and equations (3a) and (3b) into (1) to give us the full dynamics.

The dynamics of equation (1) can be divided into fast and slow phases (Pomiankowski et al. 1991; Pomiankowski and Iwasa 1993). Because *b*[*p*] << *c*[*t*], initially the selection gradient 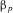 (eq. (3b)) is much smaller than 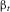 (eq. (3a)), and so during the fast dynamics phase the system can be modeled as


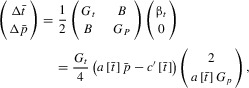
(5)

at least where 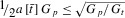 (as is likely assuming 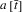 does not become too large). Thus, the system will evolve along fast dynamics evolutionary trajectories with gradients 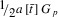 (equal to 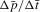), until it reaches the quasiequilibrium line 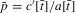 (along which 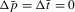 for the fast dynamics). At this point the slow dynamics phase begins, and the system converges to the origin (Appendix 2). The quasiequilibrium line will be reached assuming that the gradient of the fast dynamics evolutionary trajectories is smaller than the gradient of the quasiequilibrium line. It is only during the slow dynamics phase that the function *b*[*p*] becomes important. If the evolutionary trajectory does not converge to the quasiequilibrium line, the system fails to reach an equilibrium point but rather results in a runaway, resulting in perpetual evolution (either in the direction of increasing or decreasing 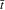 and 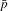).

### (a) Preference for exaggeration is less costly

Consider a cost function for preference *b*[*p*] that is nonnegative, with *b*[0] = 0 (i.e., is least costly when there is no preference), and has the property that 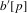 has the same sign as *p* (so that costs increase as *p* deviates from zero in either direction). We then introduce the asymmetry that *b*[*p*] < *b*[−*p*] for all *p* ≠ 0, that is, the cost of preference for exaggerated traits is less than for reduced traits. We take 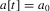 and 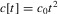, for positive constants 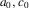.

**Figure 1:**
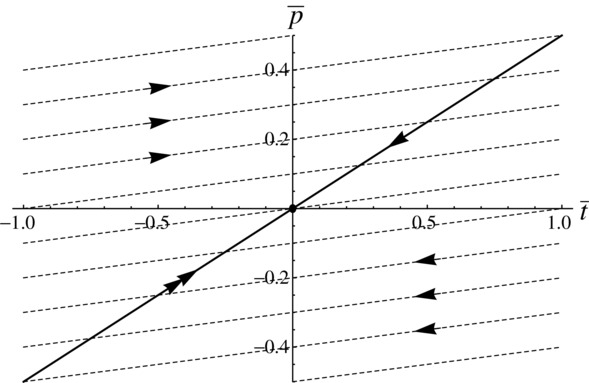
Simplified phase portrait for the case in which female preference for reduced ornaments is more costly than that for exaggerated ornaments. The system travels along the evolutionary trajectories (dashed lines) until meeting the quasiequilibrium line (thick black line). At this point, evolution proceeds along the quasiequilibrium line to the origin. The speed at which evolution proceeds along the quasiequilbrium line is more rapid from negative preference, as shown by the double arrow in the bottom left quadrant.

The function *b*[*p*] only takes effect during the slow dynamics phase, after the population has evolved onto the quasiequilibrium line 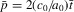. The population then proceeds along the line to the origin, which is a stable equilibrium (Appendix 2). If 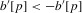, then populations with preference for reduced traits (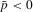) will evolve to the origin more rapidly than those with preference for exaggerated traits (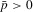), but other than this the system is unchanged. Therefore, more costly preference for reduced ornaments does not preclude evolution of reduced ornaments or create an asymmetry in the size of exaggerated or reduced traits (Fig. 1).

### (b) Ornament exaggeration is less costly

Consider a function *c*[*t*] that is nonnegative, with *c*[0] = 0, 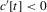 if *t* < 0 and 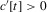 if *t* > 0. One example of asymmetry in the cost of an ornament is


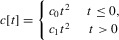


where *c*_0_ and *c*_1_ are constants, 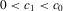, so *c*[*t*] < *c*[−*t*] for all *t* ≠ 0. The derivative of *c*[*t*] then exists and is continuous. We take 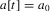, and 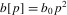, for positive constants 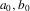, and calculate the equation of the quasiequilibrium line as being


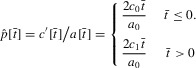
(6)

We can then show that along this line, the system evolves toward the origin, which is the only equilibrium (Appendix 2).

**Figure 2:**
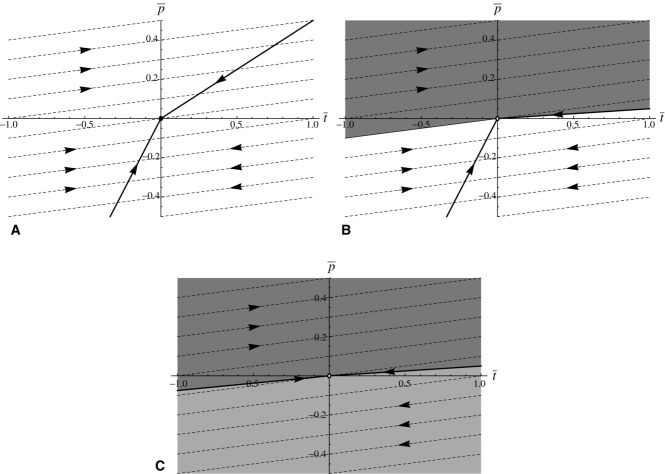
Asymmetry in the cost of ornaments in which higher costs are associated with reduced traits. (A) The case in which ¼ *a*_0_^2^ *G_p_* < *c*_1_ < *c*_0_. The quasiequilibrium line (thick black line) has a greater gradient than the fast dynamics trajectories (dashed lines). The system evolves to the stable equilibrium at the origin. (B) The case in which *c*_1_ < ¼ *a*_0_^2^ *G_p_* < *c*_0_. The gradient of the quasiequilibrium line is greater than that of the fast dynamics trajectories for 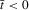 but less for 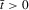. Populations within the shaded portion show runaway in the positive direction. Populations within the unshaded portion evolve to the origin, which is now an unstable equilibrium. (C) The case in which *c*_1_ < *c*_0_ < ¼ *a*_0_^2^ *G_p_*. The gradient of the quasiequilibrium line is always greater than that of the fast dynamics trajectories. Runaway now occurs in the positive direction from the darker shaded portion, and in the negative direction from the lighter shaded portion. The origin is an unstable equilibrium.

The question remaining is whether the system reaches the quasiequilibrium line. This will occur if the gradient of the function in equation (6) is greater than the gradient of the evolutionary trajectories of the fast dynamics ½*a*_0_ *G_p_*. Three cases are possible (Fig. 2; Appendix 2). The first is that ¼ *a*_0_^2^ *G_p_* < *c*_1_ < *c*_0_ and the system always evolves toward the origin (Fig. 2A). The second possibility is that *c*_1_ < ¼ *a*_0_^2^ *G_p_* < *c*_0_. The system can then either evolve to the origin or run away in the direction of exaggerated traits (Fig. 2B). The third and final possibility is that that *c*_1_ < *c*_0_ < ¼ *a*_0_^2^ *G_p_*. The system will then run away in the direction of either exaggerated or reduced traits, with the origin being an unstable equilibrium (Fig. 2C).

As a general explanation, this isn't wholly satisfactory. In order for this adaptation of the model to explain the difficulty in evolving reduced traits, we require that nature is typically as described in Figure 2B. The key value is the cost of exaggerated and reduced traits per unit deviation from the natural selection optimum. In Figure 2B, runaway occurs in the positive direction because the cost value is small enough but not in the negative direction because the cost value is too large. If the cost is too great in both directions, we are in a case like Figure 2A; if it is too small in both directions we can get runaway in either direction (Fig. 2C). In order for this to be a consistent explanation for the imbalance between exaggerated and reduced traits, then, nature needs to consistently fall into the part of parameter space corresponding to Figure 2B. Although this may happen sometimes, there is no reason to suppose it is generally the case.

### (c) Exaggerated ornaments are more efficacious signals

Consider an efficacy function *a*[*t*] such that *a*[*t*] > 0, and 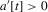, so that the efficacy of the male trait increases with size. One possible function is


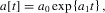


where *a*_0_ and *a*_1_ are constants, *a*_0_ > 0, *a*_1_ ≥ 0. We take *b*[*p*] = *b*_0_ *p*^2^, and *c*[*t*] = *c*_0_ *t^2^* for positive constants *b*_0_, *c*_0_. The quasiequilibrium line is then


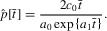
(7)

**Figure 3:**
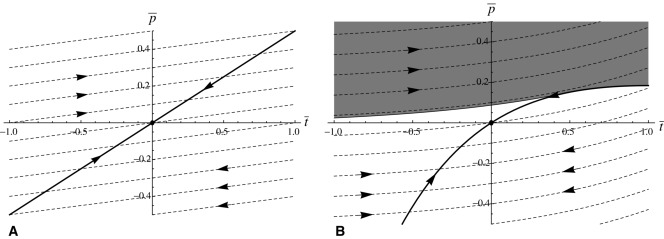
Simplified phase portraits for the case in which ornament cost is an even function but efficacy of ornament is 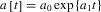. (A) 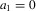. Ornament efficacy is constant for all values of *t*, and the quasiequilibrium line (solid thick line) and the fast dynamics evolutionary trajectories (dashed lines) are all straight lines. The system either evolves to a stable equilibrium at the origin as seen here, or there is runaway in both directions and the origin is unstable (not shown). (B) 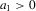. The gradient of the quasiequilibrium line decreases with 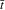, whereas the gradient of the fast dynamics trajectories increases with 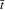. Runaway is no longer possible in the negative direction. From anywhere in the shaded portion, the system runs away in the positive direction. From anywhere in the unshaded portion, the system evolves to the stable equilibrium at the origin. Although the origin is locally stable, as *a_1_* increases its neighborhood of stability shrinks, so that smaller perturbations can result in positive runaway.

If we take *a*_1_ = 0 there is no asymmetry, as *a*[*t*] = *a*_0_ and efficacy is independent of exaggeration or reduction. In this case, the system devolves to the familiar model with quasiequilibrium line and fast dynamics trajectories being straight lines. If the gradient of the former is larger than that of the latter, then the system evolves toward the stable equilibrium at the origin (Fig. 3A); if not, the system is equally likely to runaway in the direction of exaggeration or reduction in ornament size.

However, if *a*_1_ > 0, this changes. There is now asymmetry in efficacy, which increases with trait exaggeration and decreases with trait reduction. This means that the per unit benefit through gaining more matings increases as the trait becomes exaggerated, whereas it decreases as the trait becomes reduced relative to the natural selection optimum. The quasiequilibrium line becomes concave, while the fast dynamics trajectories become convex (Fig. 3B). Runaway is always possible toward exaggeration, and never possible toward reduction (Appendix 3). The origin is locally stable, but the size of the neighborhood of points around it that have trajectories that return to the origin shrinks as *a*_1_ increases (Fig. 3B).

### (d) Combining the three hypotheses

Combining the hypothesis that preference for larger ornaments is cheaper with either of the other two hypotheses will only alter behavior along the quasiequilibrium line, as mentioned above. If larger ornaments are both cheaper and more efficacious, then the system is similar to that in which they are only more efficacious, but the size of the set of points that lead to positive runaway is increased.

## Discussion

It is a general observation that sexual traits are exaggerated rather than reduced, even though models of sexual selection predict that deviations from the natural selection optimum should occur equally in both directions (Andersson 1994; Mead and Arnold 2004; Kuijper et al. 2012). We used a survey of articles about sexual selection and sexual ornaments to confirm this impression for traits in four broad groups: color, behavior, pheromone, and morphology. This survey brought out a simple explanation for the rarity of female preference for reduced sexual color, behavior, and pheromones. These traits are typically bounded at a trait size of zero, the natural selection optimum, so negative trait size (i.e., less than the natural selection optimum) is not easily defined. For example, traits like color patches, displays during courtship, and the release of pheromones probably have natural selective optima of zero trait or behavior (they are absent from the choosy sex). This does not preclude “negative” female preference for smaller trait values if some other force maintains them (see above for examples), but it does preclude the possibility that male traits exist on a simple reduced/exaggerated scale. A possibility is that crypsis is the natural selection optimum and any deviation from this is an ornamental trait. This gives many possible directions for a sexual trait to evolve in, but it is not clear that one direction can be well defined as exaggerated and another reduced. Such problems occur with color, behavior, and pheromones.

In general, a debate about what reduction means for any color, behavior, or pheromone trait could be made and would have to take into account the specific conditions applicable to the particular example. However, there is no consistent way in which an exaggerated/reduced scale can be set up. There may be multiple dimensions of exaggeration, but there is no easy way to classify these as opposite directions on a single scale (i.e., exaggerated and reduced). So part of the answer to our initial question, why there are so few small secondary sexual characters, is that many traits used in male sexual display are limited to exaggeration and simply can not exist as reduced sexual ornaments.

This explanation also applies to certain morphological traits that are not found in females and appear to have no purpose other than in mate choice (Buchholz 1995; Mateos and Carranza 1995; Hebets and Uetz 2000; Schlupp et al. 2010). However, the majority of morphological traits that are sexual signals can be generally categorized as being exaggerated or reduced, because they are traits (such as wings, fins, or tails) that exist regardless of sexual selection. We find, overwhelmingly, that exaggerated traits are preferred, with only a few cases in which there is evidence of preference for reduced ornamental traits (Supplementary Table S1). Even when there is good evidence that males have reduced traits, explanations other than female preference for reduction seem plausible. In the well-known case of the short-tailed male golden-headed cisticola (*Cisticola exilisis*), female preference is likely to be for aerodynamic ability, which is improved by a shortened tail, rather than reduced tail size per se (Balmford et al. 2000). It is clear that female preference for reduced sexual trait size is possible but this is rarely seen in nature, except under particular contexts (e.g., Wilkinson and Reillo 1994; Johnson and Basolo 2003; Wong and Rosenthal 2006; Walling et al. 2008; Robinson et al. 2011). An explanation for this imbalance in most morphological traits is therefore required.

We investigated this using a Fisherian model of sexual selection to see if any of three potential asymmetries could favor the evolution of exaggeration. We used Fisher's model as its assumptions underpin variants of sexual selection caused by mate preference (e.g., handicap, sexual antagonism). The asymmetries relate to the three coefficients that typify models of sexual selection: the cost of preference for exaggerated or reduced traits, the cost of exaggerated or reduced traits themselves, and the signaling efficacy of exaggerated and reduced traits (*b, c*, and *a*, respectively in eq. (3). Our analysis shows that two of these do not provide general explanations for the paucity of reduced sexual traits. First, making preference for smaller ornaments more costly does not make any qualitative difference to the system (Fig. 1). If mean preference is for males with smaller ornaments, then this causes as great a runaway as would mean preference for larger ornaments, but toward reduced rather than exaggerated male traits (Fig. 1). Asymmetry in the cost of preference results in negative preference for smaller ornaments being lost quicker (Fig. 1). There is some plausibility to the idea that preferences for smaller ornaments are more costly, if discrimination between smaller objects requires more effort by the female, for instance if she needs to approach closer to males. However, the theory shows that higher preference costs do not preclude the evolution of reduced ornaments. A second possibility is that the cost of a reduced sexual trait is higher than that of an exaggerated sexual trait. This alters the slope of line of quasiequilibria, so that for a given strength of preference (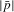) there will be less reduction (if 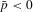) than exaggeration (if 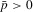; Figure 2A). However, this does not necessarily make runaway toward reduction less likely or preclude evolutionary change resulting in a quasiequilibrium preference for a reduced trait value. There are parameter values that result in indefinite runaway in the exaggerated direction but not in the reduced direction (Fig. 2B), but this is not generally the case (Figs. 2A, C). It seems highly unlikely that the cost of male ornaments falls exactly into the required part of parameter space across a broad range of taxa. It also seems somewhat paradoxical to imagine that costs are higher for reduced than exaggerated traits, because investing less in a trait seems likely to cost less as a first approximation.

Our modeling shows that the most plausible explanation is that the efficacy of sexual traits is greater in the exaggerated than in the reduced direction. Efficacy in this case is a measure of how a unit change in trait value affects the attractiveness of a male. There are many factors affecting the evolution of signaling systems (Fawcett et al. 2011), but there are good reasons to suspect that exaggerated signals consistently provide better efficacy. To be effective, signals must be detected by the receiver and distinguished from the other sensory inputs. Biophysical limits on transmission and background noise are likely to affect exaggerated ornaments less than reduced ornaments, whereas comparison between larger ornaments will be easier, particularly from distance (Endler 1993; Leichty and Grier 2006; Fawcett et al. 2007). This means that in general, efficacy is likely to increase as ornaments become larger. We have shown that if the efficacy function is sufficiently asymmetric, indefinite runaway will occur toward greater trait values but not toward reduced trait values (Fig. 3B). This finding provides mathematical support for the often-held idea that signaling traits should evolve toward greater efficacy (Endler et al. 2005) for which there are many empirical examples (e.g., Endler and Houde 1995; Cummings et al. 2003; Forsman and Hagman 2006; Cummings 2007; Endler et al. 2010).

Our results change if we alter the exact shape of the fitness functions. For example, we chose to model *a*[*t*] as an exponential function, but it is unlikely that the efficacy of a signal will increase exponentially forever. The crucial element for our result is the line of quasiequilibrium, 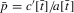. Investigating other possibilities (e.g., *a*[*t*] being a constant for *t* ≤ 0 and increasing linearly for *t* > 0) revealed qualitatively similar results (data not shown). Our conclusions point to the need for empirical evidence to establish how signal efficacy is affected by ornament size.

We also neglected some of the complexities of models of Fisher's process of sexual selection. One relevant factor is mutation bias, which has been shown to lead to the evolution of costly female mate preference for exaggerated male ornament size in the Fisher's model (Pomiankowski et al. 1991). In that analysis, it is assumed that mutation pressure is more likely to reduce the size of an ornament than to increase it, so the net effect of mutation reduces trait size. This could explain the preponderance of exaggerated over reduced sexual traits. It seems reasonable that mutations might act in a biased way to reduce exaggerated traits, but it is not obvious why mutations should be biased when ornaments are at the natural selection optimum. Consequently, the initial runaway could go in either exaggerated or reduced directions.

Another related factor that we have not directly considered is the handicap principle. We intend to examine this in future modeling efforts using already established theoretical approaches in quantitative genetics (Iwasa et al. 1991). For now, we note that the handicap process typically occurs alongside Fisher's runaway, and the distinction between these two modes of sexual selection should not be overemphasized (Iwasa and Pomiankowski 1999; Pomiankowski and Iwasa 2001; Kokko et al. 2002; Mead and Arnold 2004; Kokko et al. 2006; Kuijper et al. 2012; Chandler et al. 2013). So we expect that the qualitative results identified above will still hold. The genetic or direct benefits generated through male handicap signals will act to stabilize sexual signals (Iwasa and Pomiankowski 1999). Because handicap benefits could accrue equally to reduced as to exaggerated traits, it is not obvious *a priori* how handicap signaling could generate the far greater number of exaggerated ornaments. Another complication is that models of sexual selection predict perpetual runaway under certain conditions, as do ours here (Figs. 2, 3). This is not a biologically feasible scenario, although it is a prediction that appears in many quantitative genetic models (Mead and Arnold 2004; Kuijper et al. 2012). In reality we would expect costs of male trait exaggeration or reduction to increase at a higher rate as the ornament deviates further from the natural selection optimum, eventually causing a stop to the runaway. A generic way of incorporating this is to make the cost function *c*[*t*] a quartic rather than a quadratic (Iwasa and Pomiankowski 1995). Again, we do not think that this will qualitatively alter the logic of the explanations put forward in this article—although there may be some value in exploring this more fully if there is asymmetry in the quartic.

This article has served two purposes. First, to highlight the fact that there is a propensity for secondary sexual ornaments to be exaggerated rather than reduced, which is not consonant with existing models of sexual selection. From an empirical standpoint, we suggest that there may be more examples of reduced secondary sexual ornamentation and preference for them in the natural world than we are currently aware of. Further well-studied examples are needed to show whether there are particular ecological or perceptual reasons why reduced ornamentation has evolved. Second, we provide the grounding for theoretical explanations of this asymmetry, by showing that it is consistent with Fisher's runaway (the null model for sexual selection), if there is increased efficacy of exaggerated signals. Our model is simple, and there is a need for further work in this area to investigate the generality of our conclusions and their applicability across the whole framework of sexual selection models (Mead and Arnold 2004; Kuijper et al. 2012) and to understand whether natural systems are consistent with these theoretical ideas.

**Associate Editor: J. Hunt**

## Appendix 1. Evolution of covariance *B* between preference and trait

We follow the method of Barton and Turelli (Barton and Turelli 1991; Pomiankowski and Iwasa 1993). The contribution to the next generation of a female with preference *p* mating with a male with ornament size *t* is

where  denotes the relevant population average. After Taylor expansion of this expression around  and , and dividing by the average fitness, we have

The second term on the right-hand side indicates the intensity of directional selection on *t* due to mean female mate preference (), signaling effectiveness (), and the cost of mate choice (here represented by its rate of increase ). The third term indicates the directional selection on *p* due to the cost of choice (again represented by the rate of increase ). The fourth and fifth terms are stabilizing selection on *t* and *p*, respectively. The last term is the association of the two characters.

This gives us the following equation (following Barton and Turelli 1991; Pomiankowski and Iwasa 1993):

and from our weak selection assumption, we then get that at equilibrium,

as stated in the text.

## Appendix 2. Slow dynamics

The slow dynamics begin after convergence to the region of the quasiequilibrium line through the fast dynamics. The quasiequilibrium line has function , so the slow dynamics will have an effect for values  for some small *ε*. Then . We can rewrite equation (1) as

Thus once the system is close enough to the quasiequilibrium line (when values of *ε* are close enough to zero), evolution will proceed toward the origin, because  has the opposite sign to . The only equilibrium point is the origin.

## Appendix 3. Negative runaway is impossible if *a*_1_ ≥ 0

Assume *a*_1_ > 0. The gradient of the quasiequilibrium line (Lande 1981) is

which is decreasing in . The gradient of any fast dynamics evolutionary trajectory is , which is increasing in . Therefore, there can be only one value of  for which these two gradients are equal, which we denote , so that . Only values of *t* greater than  can lead to runaway, because it is only for these values that the gradient of the fast dynamics evolutionary trajectories is greater than that of the quasiequilibrium line. Therefore runaway is only possible in the positive direction, and never in the negative direction.

## Supporting Information

Table S1 Full details of the literature review.
