# Supplementary material for: SIGNALING EFFICACY DRIVES THE EVOLUTION OF LARGER SEXUAL ORNAMENTS BY SEXUAL SELECTION
Source: Evolution. 2013 Sep 30;68(1):216–29. doi: 10.1111/evo.12255 (PMC3920633; doi:10.1111/evo.12255)
Supplement: Table S1 — Full details of the literature review. [file evo0068-0216-sd1.docx]

**Table A1**

**Arachnids**

| Species | Ornament | Preference | Choosy sex | Reference | Notes |
| --- | --- | --- | --- | --- | --- |
|  |  |  |  |  |  |
| *Hygrolycosa rubrofasciata* | Drumming behaviour | More drumming | F | ([1](#_ENREF_1)) |  |
|  |  |  |  |  |  |
| *Schizocosa stridulans* | Leg tufts | Presence of brushes, larger brushes | F | ([2](#_ENREF_2), [3](#_ENREF_3)) |  |
|  | Courtship rate | Higher | F | ([3](#_ENREF_3)) |  |
|  |  |  |  |  |  |
| *Schizocosa crassipes* | Leg tufts | Larger brushes | F | ([2](#_ENREF_2)) |  |
|  |  |  |  |  |  |
| *Schizocosa* sp. | Leg brushes | Presence of brushes | F | ([4](#_ENREF_4)) |  |
|  |  | Good diet males | F | ([5](#_ENREF_5)) | Preference expressed only by good diet females |
|  |  |  |  |  |  |

**Insects**

| Species | Ornament | Preference | Choosy sex | Reference | Notes |
| --- | --- | --- | --- | --- | --- |
|  |  |  |  |  |  |
| *Diasemopsis meigenii* | Eyespan | Larger | F | ([6](#_ENREF_6)) |  |
|  |  |  |  |  |  |
| *Drosophila grimshawi* | Courtship behaviour | More vigourous | F | ([7](#_ENREF_7), [8](#_ENREF_8)) |  |
|  | Pheromone streak deposition | More | F | ([8](#_ENREF_8)) |  |
| *Forficula auricularia* L. | Forceps | Larger | F | ([9](#_ENREF_9)) |  |
|  |  |  |  |  |  |
| *Gryllus bimaculatus* | Call | High frequency ticks; longer duration of ticking | F | ([10](#_ENREF_10)) |  |
|  | Immunocompetence | Higher encapsulation rate | F | ([10](#_ENREF_10)) |  |
|  |  |  |  |  |  |
| *Gryllus campestris,*  *Gryllus campestris* L. | Size | Larger | F | ([11](#_ENREF_11)) |  |
|  | Age | Older | F | ([11](#_ENREF_11)) |  |
|  | Symmetry | More symmetrical | F | ([11](#_ENREF_11)) |  |
|  | Song | Pure tone song; low frequency tone | F | ([12](#_ENREF_12)) |  |
|  |  |  |  |  |  |
| *Gryllus lineaticeps* | Call | Higher chirp rate | F | ([13](#_ENREF_13)) |  |
|  |  |  |  |  |  |
| *Musca domestica* | Wings | More symmetrical | F | ([14](#_ENREF_14)) |  |
|  |  |  |  |  |  |
| *Nauphoeta cinerea* | Condition | Raised in better environmental condition | F | ([15](#_ENREF_15)) |  |
|  |  |  |  |  |  |
| *Onthophagus sagittarius* | Size | Larger | F | ([16](#_ENREF_16)) |  |
|  | Courtship rate | Higher | F | ([16](#_ENREF_16)) |  |
|  |  |  |  |  |  |
| *Pieris rapae* | Colour | Brighter white | F | ([17](#_ENREF_17)) |  |
|  |  |  |  |  |  |
| *Sabethes caneus* | Paddles on midlegs | Larger | M | ([18](#_ENREF_18)) |  |
|  |  |  |  |  |  |
|  |  |  |  |  |  |
|  |  |  |  |  |  |
| *Sepsis cynipsea* | Symmetry | More symmetrical | F | ([19](#_ENREF_19)) | Symmetrical males better able to hold on to females who try to shake them off |
| *Sigara falleni* | Foreleg palae | Larger | F | ([20](#_ENREF_20), [21](#_ENREF_21)) |  |
|  |  |  |  |  |  |
| *Stenurella melanura* | Antennae | More symmetrical | F & M | ([22](#_ENREF_22)) |  |
|  |  |  |  |  |  |
| *Teleopsis dalmanni* | Eye-stalks | Larger | F | ([23](#_ENREF_23), [24](#_ENREF_24)) |  |
|  |  |  |  |  |  |
| *Teleopsis dalmanni* | Eye-stalks | Smaller | F | ([23](#_ENREF_23)) | Females selectively bred to prefer smaller eye-span males |
|  |  |  |  |  |  |
| *Teleopsis whitei* | Eye-stalks | Larger | F | ([24](#_ENREF_24)) |  |
|  |  |  |  |  |  |
| *Teleogryllus oceanicus* | Song | Larger proportion of long chirps in songs | F | ([25](#_ENREF_25)) | Experience had a possible reversing effect on this preference |
|  |  |  |  |  |  |

**Crustaceans**

| Species | Ornament | Preference | Choosy sex | Reference | Notes |
| --- | --- | --- | --- | --- | --- |
|  |  |  |  |  |  |
| *Uca musica* | Sand hood | Various components | F | ([26](#_ENREF_26)) |  |
|  |  |  |  |  |  |
| *Uca tangeri* | Claw | Larger | F | ([27](#_ENREF_27)) |  |
|  | Courtship display | Claw position during display | F | ([27](#_ENREF_27)) |  |
|  |  |  |  |  |  |

**Fish**

| Species | Ornament | Preference | Choosy sex | Reference | Notes |
| --- | --- | --- | --- | --- | --- |
|  |  |  |  |  |  |
| *Apogon notatus* | Lower lip | Larger | F | ([28](#_ENREF_28)) |  |
|  |  |  |  |  |  |
| *Astatotilapia burtoni* | Egg-spot on tail | Fewer or absent egg-spots | F | ([29](#_ENREF_29)) |  |
|  |  |  |  |  |  |
| *Cyathopharynx furcifer* | Pelvic fin | Longer | F | ([30](#_ENREF_30)) |  |
|  |  | More symmetric | F | ([30](#_ENREF_30)) |  |
|  |  |  |  |  |  |
| *Gambusia holbrooki* | Black spots | More spots | F | ([31](#_ENREF_31)) |  |
|  |  | Fewer spots | F | ([31](#_ENREF_31)) | Females prefer spot patterns that match their local population |
|  |  |  |  |  |  |
| *Gasterosteus aculeatus* | Red colouration | Redder | F | ([32](#_ENREF_32), [33](#_ENREF_33)) |  |
|  | Size | Larger | F | ([33](#_ENREF_33)) |  |
|  | Territory quality | Better | F | ([33](#_ENREF_33)) |  |
|  | Nest | Presence of colourful material in the nest | F | ([34](#_ENREF_34)) |  |
|  | Size | Larger | F | ([33](#_ENREF_33)) |  |
|  | Spine | Symmetrical | F | ([35](#_ENREF_35)) |  |
|  |  |  |  |  |  |
| *Girardinicthys multiradiatus* | Fin size | Larger | F | ([36](#_ENREF_36)) | Fin size measured through correlates |
|  |  |  |  |  |  |
| *Gobiusculus flavescens* | Colour patches | Brighter, more colourful patches | M | ([37](#_ENREF_37), [38](#_ENREF_38)) |  |
|  |  |  |  |  |  |
|  |  |  |  |  |  |
| *Knipowitschia panizzae* | Body size | Larger | M | ([39](#_ENREF_39)) |  |
|  | Yellow belly patch | Larger | M | ([39](#_ENREF_39)) |  |
|  |  |  |  |  |  |
| *Lepomis macrochirus* | Cheek colouration | Brighter | F | ([40](#_ENREF_40)) | PCA approach |
|  | Breast colouration | Brighter | F | ([40](#_ENREF_40)) |  |
|  |  |  |  |  |  |
| *Lepomis megalotis* | Opercular flap | Longer | F | ([41](#_ENREF_41)) |  |
|  |  |  |  |  |  |
| *Pelvicachromis taeniatus* | Ventral colouration | Larger area | M | ([42](#_ENREF_42)) |  |
|  |  |  |  |  |  |
| *Phoxinus phoxinus* | Colourful markings | More colourful | F | ([43](#_ENREF_43)) | Preference for both redness and darkness in colour |
|  | Odour | Odour of males with redder bellies | F | ([44](#_ENREF_44)) | Preference for odour only in cases where females have previously been exposed to redder males |
|  |  |  |  |  |  |
| *Poecilia latipinna* | Bar markings | More bars | F | ([45](#_ENREF_45)) |  |
|  |  | More symmetrical bars | F | ([45](#_ENREF_45)) |  |
|  |  | Presence of bars | F | ([46](#_ENREF_46)) |  |
|  |  |  |  |  |  |
| *Poecilia reticulata* | Display rate | Higher | F | ([47](#_ENREF_47), [48](#_ENREF_48)) |  |
|  | Colour | Larger area of orange/red | F | ([47-53](#_ENREF_47)) |  |
|  |  | Larger area of black | F | ([49](#_ENREF_49), [51](#_ENREF_51), [54](#_ENREF_54)) |  |
|  |  | More complex colour markings | F | ([47](#_ENREF_47)) |  |
| *P. reticulata* continued | Colour continued | Duller markings | F | ([50](#_ENREF_50)) | Preference for duller males after exposure to a predator |
|  |  | Larger area of UV reflectance | F | ([55](#_ENREF_55)) |  |
|  |  | Rare colour markings | F | ([56](#_ENREF_56)) |  |
|  | Swimming speed | Higher sustained speed | F | ([47](#_ENREF_47)) |  |
|  | Dorsal fin | Longer | F | ([57](#_ENREF_57)) |  |
|  |  |  |  |  |  |
| *Poecilia sphenops* | Moustache | Presence | F | ([58](#_ENREF_58)) |  |
|  |  |  |  |  |  |
| *Rhodeus sericeus* | Colour | Brighter red | F | ([59](#_ENREF_59)) |  |
|  | Territory quality | Better | F | ([59](#_ENREF_59)) |  |
|  |  |  |  |  |  |
| *Syngnathus typhle* | Striped pattern | Higher contrast | F | ([60](#_ENREF_60)) |  |
|  |  | More ornamentation | F | ([60](#_ENREF_60)) |  |
|  | Social dominance | More dominant | F | ([61](#_ENREF_61)) |  |
|  |  |  |  |  |  |
| *Xiphophorus birchmanni* | Sword tail | Absent | F | ([62](#_ENREF_62)) |  |
|  |  | Longer dorsal fin | F | ([63](#_ENREF_63)) |  |
|  |  | Shorter dorsal fin | F | ([63](#_ENREF_63)) | Preference for both longer and shorter suggested |
|  |  |  |  |  |  |
| *Xiphophorus cortezi* | Bar markings | More symmetrical | F | ([64](#_ENREF_64), [65](#_ENREF_65)) |  |
|  |  | More bars | F | ([65](#_ENREF_65)) |  |
|  |  | Higher frequency of bars | F | ([66](#_ENREF_66)) |  |
| *X. cortezi* continued | Bar markings continued | Larger pigmented area | F | ([66](#_ENREF_66)) |  |
|  |  |  |  |  |  |
| *Xiphophorus helleri* | Size | Larger | F | ([67](#_ENREF_67)) | Possibly males seem larger because of the sword |
|  | Tail | Various components | F | ([68](#_ENREF_68)) | PCA approach |
|  |  | Longer | F | ([69](#_ENREF_69)) |  |
|  |  | Shorter | F | ([69](#_ENREF_69), [70](#_ENREF_70)) | Females prefer smaller-tailed males after exposure to a predator; or based on past experience of only short-tailed males |
|  |  |  |  |  |  |
| *Xiphophorus multilineatus* | Tail | Longer | F | ([71](#_ENREF_71)) |  |
|  |  |  |  |  |  |

**Bird**

| Species | Ornament | Preference | Choosy sex | Reference | Notes |
| --- | --- | --- | --- | --- | --- |
|  |  |  |  |  |  |
| *Acrocephalus schoenobaenus* | Song repertoire | Greater repertoire | F | ([72](#_ENREF_72)) |  |
|  | Song-flighting | More | F | ([72](#_ENREF_72)) |  |
|  | Territory size | Greater | F | ([72](#_ENREF_72)) |  |
|  |  |  |  |  |  |
| *Aethia cristatella* | Head crest | Larger | F & M | ([73](#_ENREF_73), [74](#_ENREF_74)) |  |
|  |  |  |  |  |  |
| *Agelaius phoeniceus* | Body size | Larger | F | ([75](#_ENREF_75)) |  |
|  |  |  |  |  |  |
| *Anas platyrhynchos* | Bill | Brighter (also experimentally unblackened) | F | ([76](#_ENREF_76), [77](#_ENREF_77)) |  |
|  | Plumage | Brighter (also experimentally unshaven) | F | ([76](#_ENREF_76), [77](#_ENREF_77)) |  |
|  |  |  |  |  |  |
| *Aptenodytes patagonicus* | Auricular patch | Larger | F & M | ([78](#_ENREF_78), [79](#_ENREF_79)) |  |
|  |  | Yellow colour | F & M | ([78](#_ENREF_78), [79](#_ENREF_79)) |  |
|  | Beak spot | Higher UV reflectance | F & M | ([80](#_ENREF_80)) |  |
|  |  |  |  |  |  |
| *Calipepla californica* | Crest | Larger | F | ([81](#_ENREF_81)) |  |
|  |  |  |  |  |  |
| *Callipepla gambelii* | Body mass | Larger | F | ([82](#_ENREF_82), [83](#_ENREF_83)) |  |
|  | Courtship behaviour | More | F | ([83](#_ENREF_83)) |  |
|  |  |  |  |  |  |
| *Cardinalis cardinalis* | Plumage | Redder | F & M | ([84](#_ENREF_84), [85](#_ENREF_85)) |  |
|  | Bill | Redder | F & M | ([84](#_ENREF_84)) |  |
|  |  |  |  |  |  |
| *Carpodacus mexicanus* | Plumage | Redder | F | ([86](#_ENREF_86)) |  |
|  |  |  |  |  |  |
| *Chlamydera maculata* | Courtship display | More intense display | F | ([87](#_ENREF_87)) |  |
|  |  |  |  |  |  |
| *Cisticola exilis* | Tail | Smaller | F | ([88](#_ENREF_88)) |  |
|  | Aerodynamic display | Better | F | ([88](#_ENREF_88)) |  |
|  |  |  |  |  |  |
| *Cygnus atratus* | Curled wing feathers | More feathers | F & M | ([89](#_ENREF_89)) |  |
|  |  |  |  |  |  |
| *Dendroic petechia* | Size | Larger | F | ([90](#_ENREF_90)) |  |
|  | Red streaks on breast | More streaks | F | ([90](#_ENREF_90)) |  |
|  |  |  |  |  |  |
| *Eumomota superciliosa* | Tail | Longer | F | ([91](#_ENREF_91)) |  |
|  |  |  |  |  |  |
| *Euplectes ardens* | Tail | Longer | F | ([92](#_ENREF_92), [93](#_ENREF_93)) |  |
|  |  |  |  |  |  |
| *Falco tinnunulus* | Plumage | Brighter | F | ([94](#_ENREF_94)) |  |
|  |  |  |  |  |  |
| *Ficedula hypoleuca* | Plumage | Brighter | F | ([95](#_ENREF_95)) |  |
|  |  | Darker/Blacker | F | ([96](#_ENREF_96), [97](#_ENREF_97)) |  |
|  | Wing patch | Larger | F | ([98](#_ENREF_98)) |  |
|  |  | More UV reflectant | F | ([98](#_ENREF_98)) |  |
|  | Forehead patch | Larger | F | ([96](#_ENREF_96)) |  |
|  | Tarsus | Longer | F | ([96](#_ENREF_96), [98](#_ENREF_98)) |  |
|  | Song | More versatile | F | ([98](#_ENREF_98)) |  |
|  |  |  |  |  |  |
| *Gallus gallus* | Comb | Longer | F | ([99-103](#_ENREF_99)) |  |
|  |  | Redder | F | ([99](#_ENREF_99), [100](#_ENREF_100)) |  |
|  | Iris | Redder | F | ([99](#_ENREF_99)) |  |
|  | Hackle feathers | Redder | F | ([99](#_ENREF_99)) |  |
|  | Saddle feathers | Yellower | F | ([99](#_ENREF_99)) |  |
|  | Display rate | Higher | F | ([104](#_ENREF_104), [105](#_ENREF_105)) |  |
|  | Aerobic capacity | Greater | F | ([105](#_ENREF_105)) |  |
|  | Alarm call | More frequent | F | ([106](#_ENREF_106)) |  |
|  |  |  |  |  |  |
| *Geothlypis trichas* | Black facial mask | Larger | F | ([107](#_ENREF_107)) |  |
|  | Yellow bib | Larger | F | ([108](#_ENREF_108)) |  |
|  |  |  |  |  |  |
| *Hirundo rustica rustica* | Tail | Longer | F | ([109-112](#_ENREF_109)) |  |
|  |  | More symmetrical | F | ([109](#_ENREF_109), [113](#_ENREF_113)) |  |
|  | Song rate | Higher | F | ([114](#_ENREF_114)) |  |
|  |  |  |  |  |  |
| *Hirundo rustica eythrogaster* | Ventral plumage | More colourful | F | ([115](#_ENREF_115)) |  |
| *Hirundo rustica gutturalis* | Throat plumage | More colourful | F | ([116](#_ENREF_116)) |  |
|  | Tail spots | Larger spots | F | ([116](#_ENREF_116)) |  |
|  |  |  |  |  |  |
| *Junco hyemalis* | Wings | Longer | F | ([117](#_ENREF_117)) |  |
|  | Tail | Whiter | F | ([117](#_ENREF_117)) |  |
|  |  |  |  |  |  |
| *Larosterna inca* | Moustache | Longer | F & M | ([118](#_ENREF_118)) |  |
|  |  |  |  |  |  |
| *Luscinia svecica* | Throat patch | Bluer | M | ([119](#_ENREF_119)) |  |
|  |  |  |  |  |  |
| *Malurus melanocephalus* | Nuptial plumage | Greater proportion of plumage red or black, rather than dull | F | ([120](#_ENREF_120)) | Smaller-tailed males are better in male-male competition |
|  |  |  |  |  |  |
| *Manacus vitellines* | Display | Rapidity of specific elements of display | F | ([121](#_ENREF_121)) |  |
|  |  |  |  |  |  |
| *Megadyptes antipodes* | Eye | More colourful | F & M | ([122](#_ENREF_122)) |  |
|  | Head plumage | More colourful | F & M | ([122](#_ENREF_122)) |  |
|  |  |  |  |  |  |
| *Meleagris gallopavo* | Snood | Longer | F | ([123](#_ENREF_123)) |  |
|  | Skullcap | Longer | F | ([123](#_ENREF_123)) |  |
|  |  |  |  |  |  |
| *Melopsittacus undulatus* | Plumage | Well-preened | F | ([124](#_ENREF_124), [125](#_ENREF_125)) |  |
|  |  | High UV reflectance | F | ([124](#_ENREF_124), [125](#_ENREF_125)) |  |
|  |  |  |  |  |  |
| *Oporornis formosus* | Black cap | Larger | F | ([126](#_ENREF_126)) |  |
|  |  |  |  |  |  |
| *Pachycephala pectoralis* | Breast plumage | Yellower | F | ([127](#_ENREF_127)) |  |
|  | Song repertoire | Larger | F | ([127](#_ENREF_127)) |  |
|  |  |  |  |  |  |
| *Panurus biarmicus* | Beard | Longer | F | ([128](#_ENREF_128), [129](#_ENREF_129)) |  |
| *Passer domesticus* | Tail | Longer | F & M | ([130](#_ENREF_130)) |  |
|  | Wings | Longer | F & M | ([130](#_ENREF_130)) |  |
|  | Body | Larger | F & M | ([130](#_ENREF_130)) |  |
|  |  |  |  |  |  |
| *Pavo cristatus* | Train | Mass | F | ([131](#_ENREF_131)) |  |
|  |  | Number of eyespots | F | ([132](#_ENREF_132)) |  |
|  |  | Density of eyespots | F | ([132](#_ENREF_132)) |  |
|  | Display | More | F | ([132](#_ENREF_132)) |  |
|  |  |  |  |  |  |
| *Petronia petronia* | Breast patch | Larger | M | ([133](#_ENREF_133), [134](#_ENREF_134)) |  |
|  |  |  | F | ([135](#_ENREF_135)) |  |
|  | Tail markings | Larger | F | ([136](#_ENREF_136)) |  |
|  |  |  |  |  |  |
| *Phaethon rubricauda* | Tail streamers | Longer | F & M | ([137](#_ENREF_137)) |  |
|  |  |  |  |  |  |
| *Phalacrocorax aristotelis* | Head crest | Larger | F & M | ([138](#_ENREF_138)) |  |
|  |  |  |  |  |  |
| *Phasianus colchicus* | Spur length | Longer | F | ([139](#_ENREF_139)) |  |
|  | Tail | Longer | F | ([140](#_ENREF_140)) |  |
|  | Ear tufts | Longer | F | ([140](#_ENREF_140)) |  |
|  | Black points in wattle | Present | F | ([140](#_ENREF_140)) |  |
|  | Plumage | Brighter | F | ([140](#_ENREF_140)) |  |
|  | Display behaviour | More | F | ([141](#_ENREF_141)) |  |
|  |  |  |  |  |  |
| *Phylloscopus inornatus* | Colour patch size | Larger | F | ([142](#_ENREF_142)) |  |
|  | Wing length | Longer | F | ([142](#_ENREF_142)) |  |
|  | Territory size | Larger | F | ([142](#_ENREF_142)) |  |
|  |  |  |  |  |  |
| *Phylloscopus trochilus* | Territory quality | Better | F | ([143](#_ENREF_143)) |  |
|  |  |  |  |  |  |
| *Remiz pendulinus* | Mask | Larger | F | ([144](#_ENREF_144)) |  |
| *Serinus canaria* | Colour | Higher contrast with background | F | ([145](#_ENREF_145)) |  |
|  |  |  |  |  |  |
| *Setophaga ruticilla* | Flank plumage | Brighter | F | ([146](#_ENREF_146)) |  |
|  |  |  |  |  |  |
| *Sturnus vulgaris* | Nest material | More green material | F | ([147](#_ENREF_147)) |  |
|  |  |  |  |  |  |
| *Sula nebouxii* | Blue feet | Brighter | F | ([148](#_ENREF_148)) |  |
|  |  |  | M | ([149](#_ENREF_149)) |  |
|  |  |  |  |  |  |
| *Tachycineta bicolor* | Plumage | Brightness | F | ([150](#_ENREF_150)) |  |
|  |  |  |  |  |  |
| *Taeniopygia guttata* | Chest plumage | More symmetrical | F | ([151](#_ENREF_151)) |  |
|  | Bill colour | Brighter colour | F | ([152](#_ENREF_152)) |  |
|  |  |  |  |  |  |
| *Tetrao tetrix* | Tail | Undamaged | F | ([153](#_ENREF_153)) | Only true in certain contexts |
|  |  |  |  |  |  |
| *Tyrannus forficatus* | Tail | Longer | F | ([154](#_ENREF_154)) |  |
|  |  |  |  |  |  |
| *Tyto alba* | Spotted plumage | Spottier | M | ([155](#_ENREF_155)) |  |
|  |  |  |  |  |  |
| *Vermivora chrysoptera* | Black mask | Presence | F | ([156](#_ENREF_156)) | Experimentally bleached males were rejected |
|  |  |  |  |  |  |
| *Vidua paradisaea* | Tail | Less symmetrical | F | ([157](#_ENREF_157)) |  |
|  |  | Longer | F | ([157](#_ENREF_157)) |  |
|  |  |  |  |  |  |
| *Wilsonia citrina* | Song rate | Higher | F | ([158](#_ENREF_158)) |  |
|  |  |  |  |  |  |

**Reptiles**

| Species | Ornament | Preference | Choosy sex | Reference | Notes |
| --- | --- | --- | --- | --- | --- |
|  |  |  |  |  |  |
| *Iberolacerta cyreni* | Scent markings | More of a chemical also found in prey | F | ([159](#_ENREF_159)) | Preference only shown by hungry females |
|  |  |  |  |  |  |
| *Sceloporus virgatus* | Throat markings | More intense orange | M | ([160](#_ENREF_160)) |  |
|  |  |  |  |  |  |

**Mammals**

| Species | Ornament | Preference | Choosy sex | Reference | Notes |
| --- | --- | --- | --- | --- | --- |
|  |  |  |  |  |  |
| *Macaca mulatta* | Copulation call | Presence of call | F | ([161](#_ENREF_161)) |  |
|  |  |  |  |  |  |
| *Mandrillus sphinx* | Facial colouration | Brighter | F | ([162](#_ENREF_162)) |  |
|  |  |  |  |  |  |
| *Mus musculus* | Scent | Outbred individuals | F | ([163](#_ENREF_163)) |  |
|  |  |  |  |  |  |
| *Myodes glareolus* | Condition | Better | F | ([164](#_ENREF_164)) |  |
|  |  |  |  |  |  |

**Amphibians**

| Species | Ornament | Preference | Choosy sex | Reference | Notes |
| --- | --- | --- | --- | --- | --- |
|  |  |  |  |  |  |
| *Crinia georgiana* | Call | Higher call rate | F | ([165](#_ENREF_165)) |  |
|  |  | More pulses | F | ([165](#_ENREF_165)) |  |
|  |  | Average dominant frequency | F | ([165](#_ENREF_165)) |  |
|  |  |  |  |  |  |
| *Dendrobates leucomelas* | Call | Higher call rate | F | ([166](#_ENREF_166)) |  |
|  |  | Longer chirp duration | F | ([166](#_ENREF_166)) |  |
|  |  |  |  |  |  |
| *Epipedobates tricolor* | Call | Higher call rate | F | ([166](#_ENREF_166)) |  |
|  |  | Longer chirp duration | F | ([166](#_ENREF_166)) |  |
|  |  |  |  |  |  |
| *Hyla gratiosa* | Call | Higher call rate | F | ([167](#_ENREF_167)) |  |
|  |  | Longer call duration | F | ([167](#_ENREF_167)) |  |
|  |  |  |  |  |  |
| *Physalaemus pustulosus* | Call | Presence of “chuck” | F | ([168](#_ENREF_168)) |  |
|  |  | Relative amplitude of “chuck” to “whine” | F | ([168](#_ENREF_168)) |  |
|  |  |  |  |  |  |
| *Triturus cristatus* | Dorsal crest | Larger | F | ([169](#_ENREF_169)) |  |
|  |  |  |  |  |  |

Literature Review Bibliography

1. Kotiaho J, Alatalo RV, Mappes J, Parri S. Sexual selection in a wolf spider: Male drumming activity, body size, and viability. Evolution. 1996;50(5):1977-81.

2. Hebets EA, Uetz GW. Leg ornamentation and the efficacy of courtship display in four species of wolf spider (Araneae : Lycosidae). Behavioral Ecology and Sociobiology. 2000;47(4):280-6.

3. Hebets EA, Stafstrom JA, Rodriguez RL, Wilgers DJ. Enigmatic ornamentation eases male reliance on courtship performance for mating success. Animal Behaviour. 2011;81(5):963-72.

4. Hebets EA, Vink CJ. Experience leads to preference: experienced females prefer brush-legged males in a population of syntopic wolf spiders. Behavioral Ecology. 2007;18(6):1010-20.

5. Hebets EA, Wesson J, Shamble PS. Diet influences mate choice selectivity in adult female wolf spiders. Animal Behaviour. 2008;76:355-63.

6. Cotton S, Rogers DW, Small J, Pomiankowski A, Fowler K. Variation in preference for a male ornament is positively associated with female eyespan in the stalk-eyed fly *Diasemopsis meigenii*. Proceedings of the Royal Society B-Biological Sciences. 2006;273(1591):1287-92.

7. Droney DC. Environmental influences on male courtship and implications for female choice in a lekking Hawaiian *Drosophila*. Animal Behaviour. 1996;51:821-30.

8. Droney DC, Hock MB. Male sexual signals and female choice in *Drosophila grimshawi* (Diptera : Drosophilidae). Journal of Insect Behavior. 1998;11(1):59-71.

9. Tomkins JL, Simmons LW. Female choice and manipulations of forceps size and symmetry in the earwig *Forficula auricularia* L. Animal Behaviour. 1998;56:347-56.

10. Rantala MJ, Kortet R. Courtship song and immune function in the field cricket *Gryllus bimaculatus*. Biological Journal of the Linnean Society. 2003;79(3):503-10.

11. Simmons LW. Correlates of male quality in the field cricket, *Gryllus campestris* L: Age, size, and symmetry determine pairing success in field populations. Behavioral Ecology. 1995;6(4):376-81.

12. Simmons LW, Ritchie MG. Symmetry in the songs of crickets. Proceedings of the Royal Society of London Series B-Biological Sciences. 1996;263(1375):1305-11.

13. Wagner WE, Jr., Basolo AL. The relative importance of different direct benefits in the mate choices of a field cricket. Evolution. 2007;61(3):617-22.

14. Moller AP. Sexual selection, viability selection, and developmental stability in the domestic fly *Musca domestica*. Evolution. 1996;50(2):746-52.

15. Clark DC, DeBano SJ, Moore AJ. The influence of environmental quality on sexual selection in *Nauphoeta cinerea* (Dictyoptera:Blaberidae). Behavioral Ecology. 1997;8(1):46-53.

16. Watson NL, Simmons LW. Mate choice in the dung beetle *Onthophagus sagittarius*: are female horns ornaments? Behavioral Ecology. 2010;21(2):424-30.

17. Morehouse NI, Rutowski RL. In the Eyes of the Beholders: Female Choice and Avian Predation Risk Associated with an Exaggerated Male Butterfly Color. American Naturalist. 2010;176(6):768-84.

18. South SH, Arnqvist G. Male, but not female, preference for an ornament expressed in both sexes of the polygynous mosquito *Sabethes cyaneus*. Animal Behaviour. 2011;81(3):645-51.

19. Allen GR, Simmons LW. Coercive mating, fluctuating asymmetry and male mating success in the dung fly *Sepsis cynipsea*. Animal Behaviour. 1996;52:737-41.

20. Candolin U. Opposing selection on a sexually dimorphic trait through female choice and male competition in a water boatman. Evolution. 2004;58(8):1861-4.

21. Candolin U. Why do multiple traits determine mating success? Differential use in female choice and male competition in a water boatman. Proceedings of the Royal Society of London Series B-Biological Sciences. 2005;272(1558):47-52.

22. Moller AP, Zamora-Munoz C. Antennal asymmetry and sexual selection in a cerambycid beetle. Animal Behaviour. 1997;54:1509-15.

23. Wilkinson GS, Reillo PR. Female choice response to artificial selection on an exaggerated male trait in a stalk-eyed fly. Proceedings of the Royal Society of London Series B-Biological Sciences. 1994;255(1342):1-6.

24. Wilkinson GS, Kahler H, Baker RH. Evolution of female mating preferences in stalk-eyed flies. Behavioral Ecology. 1998;9(5):525-33.

25. Bailey NW, Zuk M. Field crickets change mating preferences using remembered social information. Biology Letters. 2009;5(4):449-51.

26. Christy JH, Backwell PRY, Goshima S, Kreuter T. Sexual selection for structure building by courting male fiddler crabs: an experimental study of behavioral mechanisms. Behavioral Ecology. 2002;13(3):366-74.

27. Oliveira RF, Custodio MR. Claw size, waving display and female choice in the European fiddler crab, *Uca tangeri*. Ethology Ecology & Evolution. 1998;10(3):241-51.

28. Okuda N, Fukumori K, Yanagisawa Y. Male ornamentation and its condition-dependence in a paternal mouthbrooding cardinalfish with extraordinary sex roles. Journal of Ethology. 2003;21(2):153-9.

29. Theis A, Salzburger W, Egger B. The Function of Anal Fin Egg-Spots in the Cichlid Fish *Astatotilapia burtoni*. PloS one. 2012;7(1):e29878.

30. Karino K. Female mate preference for males having long and symmetric fins in the bower-holding cichlid *Cyathopharynx furcifer*. Ethology. 1997;103(11):883-92.

31. Bisazza A, Pilastro A. Variation of female preference for male coloration in the eastern mosquitofish *Gambusia holbrooki*. Behavior Genetics. 2000;30(3):207-12.

32. Bakker TCM. Positive genetic correlation between female preference and preferred male ornament in sticklebacks. Nature. 1993;363(6426):255-7.

33. Kraak SBM, Bakker TCM, Mundwiler B. Sexual selection in sticklebacks in the field: correlates of reproductive, mating, and paternal success. Behavioral Ecology. 1999;10(6):696-706.

34. Ostlund-Nilsson S, Holmlund M. The artistic three-spined stickleback (*Gasterosteous aculeatus*). Behavioral Ecology and Sociobiology. 2003;53(4):214-20.

35. Mazzi D, Kunzler R, Bakker TCM. Female preference for symmetry in computer-animated three-spined sticklebacks, *Gasterosteus aculeatus*. Behavioral Ecology and Sociobiology. 2003;54(2):156-61.

36. Macias Garcia C, Jimenez G, Contreras B. Correlation evidence of a sexually-selected handicap. Behavioral Ecology and Sociobiology. 1994;35(4):253-9.

37. Amundsen T, Forsgren E. Male mate choice selects for female coloration in a fish. Proceedings of the National Academy of Sciences of the United States of America. 2001;98(23):13155-60.

38. Amundsen T, Forsgren E. Male preference for colourful females affected by male size in a marine fish. Behavioral Ecology and Sociobiology. 2003;54(1):55-64.

39. Pizzolon M, Rasotto MB, Mazzoldi C. Male lagoon gobies, *Knipowitschia panizzae*, prefer more ornamented to larger females. Behavioral Ecology and Sociobiology. 2008;62(4):521-8.

40. Cogliati KM, Corkum LD, Doucet SM. Role of male spatial distribution and condition-dependent colouration on female spawning behaviour and reproductive success in bluegills. Behaviour. 2010;147(5-6):599-618.

41. Goddard K, Mathis A. Do opercular flaps of male longear sunfish (*Lepomis megalotis*) serve as sexual ornaments during female mate choice? Ethology Ecology & Evolution. 1997;9(3):223-31.

42. Baldauf SA, Bakker TCM, Kullmann H, Thuenken T. Female nuptial coloration and its adaptive significance in a mutual mate choice system. Behavioral Ecology. 2011;22(3):478-85.

43. Kekalainen J, Valkama H, Huuskonen H, Taskinen J. Multiple Sexual Ornamentation Signals Male Quality and Predicts Female Preference in Minnows. Ethology. 2010;116(10):895-903.

44. Kekalainen J, Leppanen H-R, Huuskonen H, Lai Y-T, Valkama H, Taskinen J. The information content of odour, colour and tactile cues in the mate choice of minnows. Behaviour. 2011;148(8):909-25.

45. Schluter A, Parzefall J, Schlupp I. Female preference for symmetrical vertical bars in male sailfin mollies. Animal Behaviour. 1998;56:147-53.

46. Poschadel JR, Plath M, Schlupp I. Divergent female mating preference in a clonal fish. Acta Ethologica. 2009;12(1):55-60.

47. Nicoletto PF. Female sexual response to condition-dependent ornaments in the guppy, *Poecilia-reticulata*. Animal Behaviour. 1993;46(3):441-50.

48. Kodric-Brown A, Nicoletto PF. Female choice in the guppy (*Poecilia reticulata*): the interaction between male color and display. Behavioral Ecology and Sociobiology. 2001;50(4):346-51.

49. McKinnon JS. Video mate preferences of female three-spined sticklebacks from populations with divergent male coloration. Animal Behaviour. 1995;50:1645-55.

50. Gong A, Gibson RM. Reversal of a female preference after visual exposure to a predator in the guppy, *Poecilia reticulata*. Animal Behaviour. 1996;52:1007-15.

51. Brooks R, Couldridge V. Multiple sexual ornaments coevolve with multiple mating preferences. American Naturalist. 1999;154(1):37-45.

52. Kodric-Brown A, Nicoletto PF. Age and experience affect female choice in the guppy (*Poecilia reticulata*). American Naturalist. 2001;157(3):316-23.

53. Pilastro A, Simonato M, Bisazza A, Evans JP. Cryptic female preference for colorful males in guppies. Evolution. 2004;58(3):665-9.

54. Brooks R, Caithness N. Manipulating a seemingly nonpreferred male ornament reveals a role in female choice. Proceedings of the Royal Society of London Series B-Biological Sciences. 1995;261(1360):7-10.

55. Kodric-Brown A, Johnson SC. Ultraviolet reflectance patterns of male guppies enhance their attractiveness to females. Animal Behaviour. 2002;63:391-6.

56. Zajitschek SRK, Brooks RC. Distinguishing the Effects of Familiarity, Relatedness, and Color Pattern Rarity on Attractiveness and Measuring Their Effects on Sexual Selection in Guppies (*Poecilia reticulata*). American Naturalist. 2008;172(6):843-54.

57. Karino K, Ishiwatari T, Kudo H, Sato A. Female mate preference for a costly ornament in male guppies. Behavioral Ecology and Sociobiology. 2011;65(6):1305-15.

58. Schlupp I, Riesch R, Tobler M, Plath M, Parzefall J, Schartl M. A novel, sexually selected trait in poeciliid fishes: female preference for mustache-like, rostral filaments in male *Poecilia sphenops*. Behavioral Ecology and Sociobiology. 2010;64(11):1849-55.

59. Candolin U, Reynolds JD. Sexual signaling in the European bitterling: females learn the truth by direct inspection of the resource. Behavioral Ecology. 2001;12(4):407-11.

60. Berglund A, Rosenqvist G, Bernet P. Ornamentation predicts reproductive success in female pipefish. Behavioral Ecology and Sociobiology. 1997;40(3):145-50.

61. Berglund A, Rosenqvist G. Male pipefish prefer dominant over attractive females. Behavioral Ecology. 2001;12(4):402-6.

62. Wong BBM, Rosenthal GG. Female disdain for swords in a swordtail fish. American Naturalist. 2006;167(1):136-40.

63. Robinson DM, Tudor MS, Morris MR. Female preference and the evolution of an exaggerated male ornament: the shape of the preference function matters. Animal Behaviour. 2011;81(5):1015-21.

64. Morris MR, Casey K. Female swordtail fish prefer symmetrical sexual signal. Animal Behaviour. 1998;55:33-9.

65. Morris MR. Female preference for trait symmetry in addition to trait size in swordtail fish. Proceedings of the Royal Society of London Series B-Biological Sciences. 1998;265(1399):907-11.

66. Morris MR, Elias JA, Moretz JA. Defining vertical bars in relation to female preference in the swordtail fish *Xiphophorus cortezi* (Cyprinodontiformes, Poeciliidae). Ethology. 2001;107(9):827-37.

67. Rosenthal GG, Evans CS. Female preference for swords in *Xiphophorus helleri* reflects a bias for large apparent size. Proceedings of the National Academy of Sciences of the United States of America. 1998;95(8):4431-6.

68. Basolo AL, Trainor BC. The conformation of a female preference for a composite male trait in green swordtails. Animal Behaviour. 2002;63:469-74.

69. Johnson JB, Basolo AL. Predator exposure alters female mate choice in the green swordtail. Behavioral Ecology. 2003;14(5):619-25.

70. Walling CA, Royle NJ, Lindstroem J, Metcalfe NB. Experience-induced preference for short-sworded males in the green swordtail, *Xiphophorus helleri*. Animal Behaviour. 2008;76:271-6.

71. Rosenthal GG, Martinez TYF, de Leon FJG, Ryan MJ. Shared preferences by predators and females for male ornaments in swordtails. American Naturalist. 2001;158(2):146-54.

72. Buchanan KL, Catchpole CK. Female choice in the sedge warbler, *Acrocephalus schoenobaenus*: Multiple cues from song and territory quality. Proceedings of the Royal Society of London Series B-Biological Sciences. 1997;264(1381):521-6.

73. Jones IL, Hunter FM. Mutual sexual selection in a monogamous seabird. Nature. 1993;362(6417):238-9.

74. Jones IL, Hunter FM. Experimental evidence for mutual inter- and intrasexual selection favouring a crested auklet ornament. Animal Behaviour. 1999;57:521-8.

75. Weatherhead PJ, Boag PT. Pair and extra-pair mating success relative to male quality in red-winged blackbirds. Behavioral Ecology and Sociobiology. 1995;37(2):81-91.

76. Omland KE. Female mallard mating preferences for multiple male ornaments .1. Natural variation. Behavioral Ecology and Sociobiology. 1996;39(6):353-60.

77. Omland KE. Female mallard mating preferences for multiple male ornaments .2. Experimental variation. Behavioral Ecology and Sociobiology. 1996;39(6):361-6.

78. Jouventin P, Nolan PM, Dobson FS, Nicolaus M. Coloured patches influence pairing rate in King Penguins. Ibis. 2008;150(1):193-6.

79. Pincemy G, Dobson FS, Jouventin P. Experiments on colour ornaments and mate choice in king penguins. Animal Behaviour. 2009;78(5):1247-53.

80. Nolan PM, Dobson FS, Nicolaus M, Karels TJ, McGraw KJ, Jouventin P. Mutual Mate Choice for Colorful Traits in King Penguins. Ethology. 2010;116(7):635-44.

81. Calkins JD, Burley NT. Mate choice for multiple ornaments in the California quail, *Callipepla californica*. Animal Behaviour. 2003;65:69-81.

82. Hagelin JC. A field study of ornaments, body size, and mating behavior of the Gambel's Quail. Wilson Bulletin. 2003;115(3):246-57.

83. Hagelin JC, Ligon JD. Female quail prefer testosterone-mediated traits, rather than the ornate plumage of males. Animal Behaviour. 2001;61:465-76.

84. Jawor JM, Linville SU, Beall SM, Breitwisch R. Assortative mating by multiple ornaments in northern cardinals (*Cardinalis cardinalis*). Behavioral Ecology. 2003;14(4):515-20.

85. Wolfenbarger LL. Red coloration of male northern cardinals correlates with mate quality and territory quality. Behavioral Ecology. 1999;10(1):80-90.

86. Hill GE, Nolan PM, Stoehr AM. Pairing success relative to male plumage redness and pigment symmetry in the house finch: temporal and geographic constancy. Behavioral Ecology. 1999;10(1):48-53.

87. Borgia G, Presgraves DC. Coevolution of elaborated male display traits in the spotted bowerbird: an experimental test of the threat reduction hypothesis. Animal Behaviour. 1998;56:1121-8.

88. Balmford A, Lewis MJ, Brooke MD, Thomas ALR, Johnson CN. Experimental analyses of sexual and natural selection on short tails in a polygynous warbler. Proceedings of the Royal Society of London Series B-Biological Sciences. 2000;267(1448):1121-8.

89. Kraaijeveld K, Gregurke J, Hall C, Komdeur J, Mulder RA. Mutual ornamentation, sexual selection, and social dominance in the black swan. Behavioral Ecology. 2004;15(3):380-9.

90. Yezerinac SM, Weatherhead PJ. Extra-pair mating, male plumage coloration and sexual selection in yellow warblers (*Dendroica petechia*). Proceedings of the Royal Society of London Series B-Biological Sciences. 1997;264(1381):527-32.

91. Murphy TG. Racketed tail of the male and female turquoise-browed motmot: male but not female tail length correlates with pairing success, performance, and reproductive success. Behavioral Ecology and Sociobiology. 2007;61(6):911-8.

92. Pryke SR, Andersson S. Experimental evidence for female choice and energetic costs of male tail elongation in red-collared widowbirds. Biological Journal of the Linnean Society. 2005;86(1):35-43.

93. Pryke SR, Andersson S, Lawes MJ. Sexual selection of multiple handicaps in the red-collared widowbird: Female choice of tail length but not carotenoid display. Evolution. 2001;55(7):1452-63.

94. Palokangas P, Korpimaki E, Hakkarainen H, Huhta E, Tolonen P, Alatalo RV. Female kestrels gain reproductive success by choosing brighly ornamented males. Animal Behaviour. 1994;47(2):443-8.

95. Slagsvold T, Drevon T. Female pied flycatchers trade between male quality and mating status in mate choice. Proceedings of the Royal Society of London Series B-Biological Sciences. 1999;266(1422):917-21.

96. Canal D, Potti J, Davila JA. Male phenotype predicts extra-pair paternity in pied flycatchers. Behaviour. 2011;148(5-6):691-712.

97. Galvan I, Moreno J. Variation in effects of male plumage ornaments: the case of Iberian Pied Flycatchers. Ibis. 2009;151(3):541-6.

98. Sirkia PM, Laaksonen T. Distinguishing between male and territory quality: females choose multiple traits in the pied flycatcher. Animal Behaviour. 2009;78(5):1051-60.

99. Johnson K, Thornhill R, Ligon JD, Zuk M. The direction of mothers and daughters preferences and the heritability of male ornaments in red jungle fowl (*Gallus gallus*). Behavioral Ecology. 1993;4(3):254-9.

100. Zuk M, Johnsen TS, Maclarty T. Endocrine-immune interactions, ornaments and mate choice in red jungle fowl. Proceedings of the Royal Society of London Series B-Biological Sciences. 1995;260(1358):205-10.

101. Johnsen TS, Zuk M. Repeatability of mate choice in female red jungle fowl. Behavioral Ecology. 1996;7(3):243-6.

102. Ligon JD, Kimball R, Merola-Zwartjes M. Mate choice by female red junglefowl: the issues of multiple ornaments and fluctuating asymmetry. Animal Behaviour. 1998;55:41-50.

103. Cornwallis CK, Birkhead TR. Experimental evidence that female ornamentation increases the acquisition of sperm and signals fecundity. Proceedings of the Royal Society B-Biological Sciences. 2007;274(1609):583-90.

104. Zuk M, Popma SL, Johnsen TS. Male courtship displays, ornaments and female mate choice in captive red jungle fowl. Behaviour. 1995;132:821-36.

105. Chappell MA, Zuk M, Johnsen TS, Kwan TH. Mate choice and aerobic capacity in red junglefowl. Behaviour. 1997;134:511-29.

106. Wilson DR, Bayly KL, Nelson XJ, Gillings M, Evans CS. Alarm calling best predicts mating and reproductive success in ornamented male fowl, *Gallus gallus*. Animal Behaviour. 2008;76:543-54.

107. Tarof SA, Dunn PO, Whittingham LA. Dual functions of a melanin-based ornament in the common yellowthroat. Proceedings of the Royal Society B-Biological Sciences. 2005;272(1568):1121-7.

108. Dunn PO, Whittingham LA, Freeman-Gallant CR, DeCoste J. Geographic variation in the function of ornaments in the common yellowthroat *Geothlypis trichas*. Journal of Avian Biology. 2008;39(1):66-72.

109. Moller AP. Morphology and sexual selection in the barn swallow *Hirundo-rustica* in Chernobyl, Ukraine. Proceedings of the Royal Society of London Series B-Biological Sciences. 1993;252(1333):51-7.

110. Delope F, Moller AP. Female reproductive effort depends on the degree of ornamentation of their mates. Evolution. 1993;47(4):1152-60.

111. Moller AP. Sexual selection in the barn swaller *Hirundo-rustica* 3. Female tail ornaments. Evolution. 1993;47(2):417-31.

112. Saino N, Primmer CR, Ellegren H, Moller AP. An experimental study of paternity and tail ornamentation in the barn swallow (*Hirundo rustica*). Evolution. 1997;51(2):562-70.

113. Moller AP. Female preference for apparently symmetrical male sexual ornaments in the barn swallow *Hirundo-rustica*. Behavioral Ecology and Sociobiology. 1993;32(6):371-6.

114. Moller AP, Saino N, Taramino G, Galeotti P, Ferrario S. Paternity and multiple signaling: Effects of a secondary sexual character and song on paternity in the barn swallow. American Naturalist. 1998;151(3):236-42.

115. Safran RJ, McGraw KJ. Plumage coloration, not length or symmetry of tail-streamers, is a sexually selected trait in North American barn swallows. Behavioral Ecology. 2004;15(3):455-61.

116. Hasegawa M, Arai E, Watanabe M, Nakamura M. Mating advantage of multiple male ornaments in the Barn Swallow *Hirundo rustica gutturalis*. Ornithological Science. 2010;9(2):141-8.

117. McGlothlin JW, Parker PG, Nolan V, Ketterson ED. Correlational selection leads to genetic integration of body size and an attractive plumage trait in dark-eyed juncos. Evolution. 2005;59(3):658-71.

118. Velando A, Lessells CM, Marquez JC. The function of female and male ornaments in the Inca Tern: evidence for links between ornament expression and both adult condition and reproductive performance. Journal of Avian Biology. 2001;32(4):311-8.

119. Amundsen T, Forsgren E, Hansen LTT. On the function of female ornaments: male bluethroats prefer colourful females. Proceedings of the Royal Society of London Series B-Biological Sciences. 1997;264(1388):1579-86.

120. Karubian J, Swaddle JP, Varian-Ramos CW, Webster MS. The relative importance of male tail length and nuptial plumage on social dominance and mate choice in the red-backed fairy-wren *Malurus melanocephalus*: evidence for the multiple receiver hypothesis. Journal of Avian Biology. 2009;40(5):559-68.

121. Barske J, Schlinger BA, Wikelski M, Fusani L. Female choice for male motor skills. Proceedings of the Royal Society B-Biological Sciences. 2011;278(1724):3523-8.

122. Massaro M, Davis LS, Darby JT. Carotenoid-derived ornaments reflect parental quality in male and female yellow-eyed penguins (*Megadyptes antipodes*). Behavioral Ecology and Sociobiology. 2003;55(2):169-75.

123. Buchholz R. Female choice, parasite load and male ornamentation in wild turkeys. Animal Behaviour. 1995;50:929-43.

124. Griggio M, Hoi H, Pilastro A. Plumage maintenance affects ultraviolet colour and female preference in the budgerigar. Behavioural Processes. 2010;84(3):739-44.

125. Zampiga E, Hoi H, Pilastro A. Preening, plumage reflectance and female choice in budgerigars. Ethology Ecology & Evolution. 2004;16(4):339-49.

126. Parker TH, Stansberry BM, Becker CD, Gipson PS. Do melanin- or carotenoid-pigmented plumage ornaments signal condition and predict pairing success in the Kentucky Warbler? Condor. 2003;105(4):663-71.

127. van Dongen WFD, Mulder RA. Multiple ornamentation, female breeding synchrony, and extra-pair mating success of golden whistlers (*Pachycephala pectoralis*). Journal of Ornithology. 2009;150(3):607-20.

128. Hoi H, Griggio M. Dual Utility of a Melanin-Based Ornament in Bearded Tits. Ethology. 2008;114(11):1094-100.

129. Hoi H, Griggio M. Is female mate preference based on the interaction between static and dynamic signals in bearded reedlings? Ethology Ecology & Evolution. 2011;23(2):171-8.

130. Moreno-Rueda G. Sexual size dimorphism and assortative mating for morphological traits in *Passer domesticus*. Journal of Ethology. 2006;24(3):227-30.

131. Petrie M, Williams A. Peahens lay more eggs for peacocks with larger trains. Proceedings of the Royal Society of London Series B-Biological Sciences. 1993;251(1331):127-31.

132. Loyau A, Jalme MS, Sorci G. Intra-and intersexual selection for multiple traits in the peacock (*Pavo cristatus*). Ethology. 2005;111(9):810-20.

133. Griggio M, Valera F, Casas A, Pilastro A. Males prefer ornamented females: a field experiment of male choice in the rock sparrow. Animal Behaviour. 2005;69:1243-50.

134. Griggio M, Devigili A, Hoi H, Pilastro A. Female ornamentation and directional male mate preference in the rock sparrow. Behavioral Ecology. 2009;20(5):1072-8.

135. Griggio M, Serra L, Licheri D, Monti A, Pilastro A. Armaments and ornaments in the rock sparrow: a possible dual utility of a carotenoid-based feather signal. Behavioral Ecology and Sociobiology. 2007;61(3):423-33.

136. Griggio M, Valera F, Casas-Criville A, Hoi H, Barbosa A. White tail markings are an indicator of quality and affect mate preference in rock sparrows. Behavioral Ecology and Sociobiology. 2011;65(4):655-64.

137. Veit AC, Jones IL. Function of tail streamers of Red-tailed Tropicbirds (*Phaethon rubricauda*) as inferred from patterns of variation. Auk. 2003;120(4):1033-43.

138. Daunt F, Monaghan P, Wanless S, Harris MP. Sexual ornament size and breeding performance in female and male European Shags *Phalacrocorax aristotelis*. Ibis. 2003;145(1):54-60.

139. Grahn M, Vonschantz T. Fashion and age in pheasants - age-differences in mate choice. Proceedings of the Royal Society of London Series B-Biological Sciences. 1994;255(1344):237-41.

140. Mateos C, Carranza J. Female choice for morphological features of male ring-necked pheasants. Animal Behaviour. 1995;49(3):737-48.

141. Mateos C, Carranza J. Effects of male dominance and courtship display on female choice in the ring-necked pheasant. Behavioral Ecology and Sociobiology. 1999;45(3-4):235-44.

142. Marchetti K. The evolution of multiple male traits in the yellow-browed leaf warbler. Animal Behaviour. 1998;55:361-76.

143. Gil D, Slater PJB. Multiple song repertoire characteristics in the willow warbler (*Phylloscopus trochilus*): Correlations with female choice and offspring viability. Behavioral Ecology and Sociobiology. 2000;47(5):319-26.

144. Pogany A, Szekely T. Female choice in the penduline tit *Remiz pendulinus*: the effects of nest size and male mask size. Behaviour. 2007;144:411-27.

145. Heindl M, Winkler H. Female canaries (*Serinus canaria*) associate more with males that contrast strongly against the background. Ethology. 2003;109(3):259-71.

146. Kappes PJ, Stutchbury BJM, Woolfenden BE. The relationship between carotenoid-based coloration and pairing, within- and extra-pair mating success in the American redstart. Condor. 2009;111(4):684-93.

147. Brouwer L, Komdeur J. Green nesting material has a function in mate attraction in the European starling. Animal Behaviour. 2004;67:539-48.

148. Torres R, Velando A. A dynamic trait affects continuous pair assessment in the blue-footed booby, *Sula nebouxii*. Behavioral Ecology and Sociobiology. 2003;55(1):65-72.

149. Torres R, Velando A. Male preference for female foot colour in the socially monogamous blue-footed booby, *Sula nebouxii*. Animal Behaviour. 2005;69:59-65.

150. Bitton P-P, O'Brien EL, Dawson RD. Plumage brightness and age predict extrapair fertilization success of male tree swallows, *Tachycineta bicolor*. Animal Behaviour. 2007;74:1777-84.

151. Swaddle JP, Cuthill IC. Female zebra finches prefer males with symmetrical chest plumage. Proceedings of the Royal Society of London Series B-Biological Sciences. 1994;258(1353):267-71.

152. Blount JD, Metcalfe NB, Birkhead TR, Surai PF. Carotenoid modulation of immune function and sexual attractiveness in zebra finches. Science. 2003;300(5616):125-7.

153. Hoglund J, Alatalo RV, Lundberg A, Ratti O. Context-dependent effects of tail-ornament damage on mating success in black grouse. Behavioral Ecology. 1994;5(2):182-7.

154. Regosin JV, Pruett-Jones S. Sexual selection and tail-length dimorphism in Scissor-tailed Flycatchers. Auk. 2001;118(1):167-75.

155. Roulin A. Nonrandom pairing by male barn owls (*Tyto alba*) with respect to a female plumage trait. Behavioral Ecology. 1999;10(6):688-95.

156. Leichty ER, Grier JW. Importance of facial pattern to sexual selection in golden-winged Warbler (*Vermivora chrysoptera*). Auk. 2006;123(4):962-6.

157. Oakes EJ, Barnard P. Fluctuating asymmetry and mate choice in paradise whydahs, *Vidua-paradisiae* - an experimental manipulation. Animal Behaviour. 1994;48(4):937-43.

158. Chiver I, Stutchbury BJM, Morton ES. Do male plumage and song characteristics influence female off-territory forays and paternity in the hooded warbler? Behavioral Ecology and Sociobiology. 2008;62(12):1981-90.

159. Martin J, Lopez P. Female sensory bias may allow honest chemical signaling by male Iberian rock lizards. Behavioral Ecology and Sociobiology. 2008;62(12):1927-34.

160. Weiss SL. Reproductive signals of female lizards: Pattern of trait expression and male response. Ethology. 2002;108(9):793-813.

161. Hauser MD. Rhesus-monkey copulation calls - honest signals for female choice. Proceedings of the Royal Society of London Series B-Biological Sciences. 1993;254(1340):93-6.

162. Setchell JM. Do female mandrills prefer brightly colored males? International Journal of Primatology. 2005;26(4):715-35.

163. Ilmonen P, Stundner G, Thoss M, Penn DJ. Females prefer the scent of outbred males: good-genes-as-heterozygosity? Bmc Evolutionary Biology. 2009;9.

164. Lopuch S, Radwan J. Condition dependence of sexual attractiveness in the bank vole. Behavioral Ecology and Sociobiology. 2009;63(3):339-44.

165. Smith MJ, Roberts JD. An experimental examination of female preference patterns for components of the male advertisement call in the quacking frog, *Crinia georgiana*. Behavioral Ecology and Sociobiology. 2003;55(2):144-50.

166. Forsman A, Hagman M. Calling is an honest indicator of paternal genetic quality in poison frogs. Evolution. 2006;60(10):2148-57.

167. Burke EJ, Murphy CG. How female barking treefrogs, *Hyla gratiosa*, use multiple call characteristics to select a mate. Animal Behaviour. 2007;74:1463-72.

168. Baugh AT, Ryan MJ. The relative value of call embellishment in tungara frogs. Behavioral Ecology and Sociobiology. 2011;65(2):359-67.

169. Malmgren JC, Enghag M. Female preference for male dorsal crests in great crested newts (*Triturus cristatus*). Ethology Ecology & Evolution. 2008;20(1):71-80.
